# Chemical Genetics Screen Identifies Epigenetic Mechanisms Involved in Dopaminergic and Noradrenergic Neurogenesis in Zebrafish

**DOI:** 10.3389/fgene.2020.00080

**Published:** 2020-02-25

**Authors:** Markus Westphal, Pooja Sant, Alexander-Thomas Hauser, Manfred Jung, Wolfgang Driever

**Affiliations:** ^1^ Developmental Biology, Faculty of Biology, Institute Biology 1, Albert Ludwigs University Freiburg, Freiburg, Germany; ^2^ CIBSS and BIOSS—Centres for Biological Signalling Studies, University of Freiburg, Freiburg, Germany; ^3^ Spemann Graduate School of Biology and Medicine (SGBM), University of Freiburg, Freiburg, Germany; ^4^ Chemical Epigenetics Group, Institute of Pharmaceutical Sciences, Albert Ludwigs University Freiburg, Freiburg, Germany; ^5^ CIBSS—Centre for Integrative Biological SignallingStudies, University of Freiburg, Freiburg, Germany

**Keywords:** dopaminergic neuron, epigenetic mechanism, chemical genetics, zebrafish, noradrenergic neuron

## Abstract

The cell type diversity and complexity of the nervous system is generated by a network of signaling events, transcription factors, and epigenetic regulators. Signaling and transcriptional control have been easily amenable to forward genetic screens in model organisms like zebrafish. In contrast, epigenetic mechanisms have been somewhat elusive in genetic screens, likely caused by broad action in multiple developmental pathways that masks specific phenotypes, but also by genetic redundancies of epigenetic factors. Here, we performed a screen using small molecule inhibitors of epigenetic mechanisms to reveal contributions to specific aspects of neurogenesis in zebrafish. We chose development of dopaminergic and noradrenergic neurons from neural progenitors as target of epigenetic regulation. We performed the screen in two phases: First, we tested a small molecule inhibitor library that targets a broad range of epigenetic protein classes and mechanisms, using expression of the dopaminergic and noradrenergic marker *tyrosine hydroxylase* as readout. We identified 10 compounds, including HDAC, Bromodomain and HAT inhibitors, which interfered with dopaminergic and noradrenergic development in larval zebrafish. In the second screening phase, we aimed to identify neurogenesis stages affected by these 10 inhibitors. We analyzed treated embryos for effects on neural stem cells, growth progression of the retina, and apoptosis in neural tissues. In addition, we analyzed effects on *islet1* expressing neuronal populations to determine potential selectivity of compounds for transmitter phenotypes. In summary, our targeted screen of epigenetic inhibitors identified specific compounds, which reveal chromatin regulator classes that contribute to dopaminergic and noradrenergic neurogenesis *in vivo*.

## Introduction

Dopaminergic (DA) neurons develop under control of complex genetic programs, and need to be maintained lifelong to sustain proper brain function (Blaess and Ang, 2015). There is strong interest in mechanisms of stable DA differentiation to develop cell replacement or regenerative therapies for neurodegenerative loss of dopaminergic neurons (Arenas et al., 2015; Barker et al., 2017). The establishment and maintenance of DA, or in general, cellular differentiation programs depends on chromatin dynamics and regulation of gene expression to specify and constrain developmental trajectories (Hsieh and Zhao, 2016; Perino and Veenstra, 2016). The epigenetic state of a cell, as defined by chromatin epigenetic modifications and chromatin structure, initiates and maintains cell-type specific transcriptional programs. Chromatin regulators comprise several families of chromatin-binding proteins and of enzymes that add or remove covalent modifications to histones (Arrowsmith et al., 2012). The dynamic and reversible nature of chromatin states is important for proper gene regulation in development (Yu et al., 1995; Gregg et al., 2003; Cunliffe, 2004; Lim et al., 2009). However, a detailed characterization of chromatin regulator function in neural development remains elusive, as early phenotypes in epigenetic mutants, as well as redundant gene functions, may obstruct analysis of late organogenesis functions of epigenetic factors.

Recently, small molecule compounds targeting chromatin regulators have been developed, mainly motivated by increased understanding of chromatin misregulation during cancer progression. These small molecule compounds act by blocking the activity of distinct chromatin regulator protein families and thereby alter the chromatin state of a cell (Arrowsmith et al., 2012). Thus, small molecule inhibitors provide a means to identify epigenetic mechanisms in development, even when the epigenetic factors themselves may be encoded redundantly, or broadly required during earlier phases of development (Kubicek et al., 2012). Chemical genetics exploits such active compounds to identify molecular mechanisms contributing to developmental decisions (Yeh and Crews, 2003). Zebrafish provides a highly amenable system for chemical genetic screens to assay processes that small molecule compounds might influence *in vivo* (Peterson et al., 2000; North et al., 2007; Kokel et al., 2010). Successful chemical genetic screens performed on zebrafish embryos also uncovered novel contributions of chromatin factors during vertebrate development (Cao et al., 2009; Early et al., 2018).

Transcriptional mechanisms of cell fate acquisition in the developing nervous system are tightly linked to chromatin regulation (Yao et al., 2016). Polycomb (PcG) and Trithorax group (TrxG) proteins control neuronal cell fate transitions and proliferation in embryonic and adult neural stem cells (NSC) (Fasano et al., 2007; Hirabayashi et al., 2009; Lim et al., 2009). Furthermore, histone deacetylases (HDACs) sustain core neurogenic transcriptional profiles during neurogenesis *in vivo* (Cunliffe, 2004). However, chromatin regulator functions in initiation and maintenance of specific neuronal lineage programs are not fully understood. To gain a better understanding is important, because mutations in chromatin regulators have been linked to a variety of neurodevelopmental and neurodegenerative disorders as well as psychiatric diseases in humans (Jakovcevski and Akbarian, 2012).

Parkinson's disease, a prevalent neurodegenerative disorder, predominantly affects dopaminergic (DA) neurons. DA neurons form a major neuromodulatory system in the brain controlling the regulation of homeostasis, mood, cognition and motor control. They develop in stereotypic positions in the forebrain and ventral midbrain in mammals (Bjorklund and Dunnett, 2007). Because DA neurons in the substantia nigra (SN) of the ventral midbrain are severely affected in Parkinson´s disease, transcriptional and epigenetic mechanisms contributing to differentiation and survival of these neurons have been intensely studied (Harrison and Dexter, 2013; van Heesbeen et al., 2013). Chromatin regulators act during different stages of midbrain DA neuron development to specify transcriptional landscapes of DA progenitors and neurons. For example, in DA progenitors, HDACs specifically repress transcriptional networks permitting midbrain DA neuron development (Jacobs et al., 2009). This is accomplished by the transcription factor *Pitx3* interacting with *Nurr1* to guide development of midbrain DA neurons by releasing SMRT-HDAC repressive complexes from the promoters of *Nurr1* target genes. Further, the PcG protein Ezh2 is required for the differentiation of midbrain DA progenitors into mature DA neurons, and later maintains post-mitotic DA neuron identity in adult mice (Wever et al., 2018).

We and others have used the zebrafish model to better understand fundamental aspects of DA systems development. In zebrafish, DA cell clusters have been identified exclusively within the forebrain (Holzschuh et al., 2001; Kaslin and Panula, 2001; Rink and Wullimann, 2002). DA neurons in the zebrafish forebrain reside in the olfactory bulb, subpallium, retina, preoptic area, pretectum, ventral diencephalon, and hypothalamus, but are absent from the midbrain. DA and noradrenergic (NA) neurons contain neuromodulators which both derive from tyrosine, and together belong to the class of catecholaminergic (CA) neurons. Brain NA neurons reside exclusively within the locus coeruleus (LC) and medulla oblongata (MO) of the hindbrain. Despite the large evolutionary distance between zebrafish and mammals, important anatomical, molecular and functional homologies between zebrafish and mammalian forebrain DA groups exist (Ryu et al., 2007; Lohr et al., 2009; Filippi et al., 2012). Control of DA neurogenesis by signaling and transcription factor networks has been extensively studied in both rodent and zebrafish models (Ang, 2006; Yamamoto and Vernier, 2011; Hegarty et al., 2013). In zebrafish, signaling pathways and transcription factors have been shown to contribute to DA differentiation from neural stem cells (NSCs) (Holzschuh et al., 2003; Filippi et al., 2007; Mahler et al., 2010; Filippi et al., 2012). However, epigenetic control of zebrafish DA development is unknown.

To identify chromatin regulatory mechanisms that function during DA neurogenesis, we performed a chemical genetics screen in zebrafish embryos using small molecule inhibitors of chromatin regulators. We compared *in situ* in treated embryos effects on distinct neuron types, specifically, *tyrosine hydroxylase (th)* expressing DA and NA neurons, as well as *islet1 (isl1)* expressing neurons. We selected 32 small molecule inhibitors that target a broad range of chromatin regulator classes and mechanisms. We identified small molecule inhibitors targeting histone deacetylases (HDACs), acetyl reader Bromodomain proteins and histone acetyltransferases (HATs) to affect DA neuron differentiation. Subsequent analyses characterized neurogenesis stages during which the small molecule exposure might interfere. We found that distinct chromatin regulators differentially affect neural stem and progenitor cell maintenance and neurogenesis in specific DA neuron groups. Our chemical genetics screen provides novel leads to chromatin regulatory mechanisms that contribute to DA neurogenesis *in vivo*.

## Material and Methods

### Zebrafish Husbandry and Strains

Zebrafish care and breeding were performed under standard conditions (Westerfield, 2000) (http://zfin.org). Zebrafish embryos were incubated at 28.5°C in E3 medium (5 mM NaCl, 0.17 mM KCl, 0.33 mM CaCl_2_, 0.33 mM MgSO_4_) containing 0.2 mM phenylthiourea to avoid melanin pigmentation. Embryos were staged according to Kimmel et al., 1995. All work on zebrafish embryos and adults were in accordance with German laws for animal care under permission from the Regierungspräsidium Freiburg.

We used the zebrafish AB/TL wildtype strain in all experiments. For the analysis of Islet1 positive cranial motor neurons, we used the transgenic Tg(*islet1:GFP*)*^rw0^* reporter line (Higashijima et al., 2000). For experiments, heterozygous Tg(*islet1:GFP*)*^rw0^* fish were outcrossed to AB/TL fish.

### Epigenetic Small Molecule Compounds

**Supplementary Figure 1** lists all small molecule compounds used in the screens. Selected epigenetic small molecule compounds were obtained from the Collaborative Research Centre 992 Medical Epigenetics (CRC992 MEDEP; Laboratory of Manfred Jung) at University of Freiburg, and from the Structural Genomics Consortium (SGC). Additionally, the following chemical compounds were obtained commercially: Bromosporine (Sigma #SML0992), Ex-527 (Selleckchem #S1541), MM-102 (Selleckchem #S7265), Mocetinostat (Selleckchem #S1122), OICR9429 (Tocris # 5267), PFI-3 (Selleckchem #S7315), UNC0631 (Selleckchem, S7610). All small molecule compounds were dissolved in DMSO (99.5%, PanReac AppliChem #A3672) at 10 mM concentration. For experiments, the 10 mM stock was diluted with E3 medium to obtain the desired working stock concentration of 60 µM containing 1% DMSO, which was used to prepare the dilution series. The γ-secretase inhibitor DAPT (increased DA neurogenesis; (Mahler et al., 2010); Sigma, #D5942) and the prodrug neurotoxin MPTP (neurotoxic to DA neurons; (Sallinen et al., 2009); Sigma #M0896) were used as positive controls for drug delivery into embryos and dopaminergic neurons.

### Treatment of Embryos With Small Molecules

Small molecule compounds were screened at a concentration range of 30, 10, 3, and 1 µM in E3 medium containing 1% DMSO. These compounds were tested alongside 1% DMSO as vehicle control and 100 µM DAPT and 1 mM MPTP as positive controls for pharmacological effects on DA neurogenesis and neuron survival respectively. Zebrafish embryos at desired developmental stages from age-matched separate spawnings were pooled and arrayed into 24-well plates with 12 embryos per well. Five hundred microliters of E3 medium containing 0.2 mM phenylthiourea (PTU) and 1% DMSO was added to each well. Working solutions of small molecule compounds were prepared in a separate 24-well plate. A serial dilution was prepared at 60, 20, 6, and 2 µM in E3 medium/1× PTU/1% DMSO. Five hundred microliters of each working solution was added to the respective well containing 500 µl E3 medium and the embryos. The final volume of 1,000 µl in each well corresponded to compound concentrations of 30, 10, 3, and 1 µM. Screening assay controls for drug delivery were used at active concentrations resulting in impaired DA or NA neurogenesis or survival as described before (Sallinen et al., 2009; Mahler et al., 2010). Working solutions of DAPT and MPTP were prepared at 200 µM and 2 mM respectively. Addition of 500 µl of each working solution to the embryos in 500 µl E3 medium/1× PTU/1% DMSO yielded a final concentration of 100 µM DAPT and 1 mM MPTP. Embryos were incubated at 28.5°C until 72 h post fertilization (hpf) and the small molecule solution was then washed out by five E3 medium washes. Embryos were incubated for an additional 24 h in E3 medium/1× PTU at 28.5°C. Embryo morphology was assessed and documented at 96 hpf using a stereomicroscope. For subsequent immunohistochemistry, embryos from each treatment were transferred into separate 2.0 ml reaction tubes. Zebrafish embryos at 96 hpf were fixed in 4% PFA in PBST (1xPBS, 0.1% Tween-20), dehydrated in increasing concentrations of methanol/PBST and stored in absolute methanol at -20°C.

### Whole-Mount *In-Situ* Hybridization

Alkaline phosphatase based standard colorigenic whole mount *in situ* hybridization (WISH) was performed based on established protocols (Holzschuh et al., 2003). The following digoxigenin-labeled antisense riboprobes were generated: *sox2* (Chapouton et al., 2006) and *th* (Holzschuh et al., 2003). Antisense probes were *in vitro* transcribed respectively with T7 or T3 RNA polymerase along with DIG labeling mix (Roche). Fixed embryos were rehydrated in decreasing concentrations of methanol/PBST and transferred to PBST. Embryos at 96 hpf were treated with proteinase K (10 µg/ml in PBST) for 1 h at room temperature (RT). Proteinase K permeabilization was stopped by postfixing the embryos in 4% PFA/PBST for 20 min at RT. After three PBST washes, embryos were pre-hybridized in Hyb-Mix (50% formamide, 5x SSC, 50 µg/ml heparin, 0.1% Tween-20, 5 mg/ml torula RNA) for 2 h at 65°C. Hyb mix was replaced by 300 µl Hyb mix containing 50 ng digoxygenin labeled antisense probe. Hybridization was performed overnight at 65°C. High-stringency washes in 50% formamide/5× SSCT (0.30 M NaCl, 0.030 M trisodium citrate, pH 7.0), 2× SSCT buffer and 0.2× SSCT buffer were performed at 65°C. Embryos were washed twice in PBST and then transferred to WISH blocking solution (2% NGS, 2 mg/ml BSA in PBST) for 2 h at RT. The embryos were then incubated in anti-Digoxygenin alkaline phosphatase conjugated Fab fragment (Roche) at a 1:5,000 dilution in PBST overnight at 4°C. Excess antibody was washed out by five washes in PBST at RT. Prior to staining, embryos were transferred for 15 min to 100 mM Tris-HCl (pH 9.5) followed by incubation in 1x staining buffer (100 mM NaCl, 50 mM MgCl_2_, 100 mM Tris HCl pH 9.5, 1 mM levamisole, 0.1% Tween-20) for 15 min at RT. BCIP/NBT was used as a chromogenic substrate for alkaline phosphatase (160 ng/ml BCIP and NBT in staining buffer). Staining was performed in 24-well plates for 1 ½ h at RT in the dark. Staining reaction was stopped by three washes with WISH stop solution (PBST/EDTA, pH 5.5). Stained embryos were cleared and stored in 100% glycerol.

### Immunohistochemistry

Peroxidase based colorigenic immunohistochemistry was used to detect Islet1:GFP transgene expression in Tg(*islet1:GFP*) embryos and to detect pH3 immunoreactive cells in WT embryos. Following fixation but prior to permeabilization, embryos were incubated in 1% H_2_O_2_/PBST solution to inactivate endogenous peroxidase activity. Embryos at 96 hpf were treated with proteinase K (10 µg/ml in PBST) for 1 h at RT and the reaction was stopped by postfixing the embryos in 4% PFA/PBST for 20 min. After several washes in PBSTD (1× PBS, 0.1% Tween-20, 1% DMSO) embryos were incubated in blocking reagent (2 mg/ml BSA, 5% NGS, 1% Boehringer Blocking Reagent in PBSTD) for 2 h at RT. Primary chicken anti-GFP antibody (Invitrogen) or rabbit anti-pH3 antibody (Stern et al., 2005) (Sigma/Millipore; 06-570) were used at a 5 µg/ml dilution in blocking reagent. Embryos were incubated in the presence of primary antibody overnight at 4°C. Ten washes were performed for 15 min each in PBSTD. Secondary biotinylated goat anti chicken or biotinylated goat anti rabbit sera (Vector Laboratories, Burlingame USA) were used at a 1:1,000 dilution in blocking reagent and embryos were incubated in secondary antibody overnight at 4°C. Antigen detection was performed using the Vectastain Elite ABC Kit (Vector Laboratories, Burlingame USA) according to the manufacturer's recommendation. Diaminobenzidine (DAB) was used as a chromogenic substrate for the peroxidase. Embryos were pre-equilibrated for 15 minutes in DAB solution (0.2 mg/ml DAB in PBSTD). Staining was performed in DAB solution containing 0.3% H_2_O_2_ for 5 min in the dark. Stained embryos were washed several times in PBST and transferred to 100% glycerol for clearing and storing.

### TUNEL Staining

Detection of apoptotic cell death by TUNEL staining was performed using the Chemicon ApopTag *In Situ* Apoptosis Detection Kit (Millipore Bioscience Research). The protocol was modified from (Abdelilah et al., 1996). Embryos at 96 hpf were permeabilized in proteinase K (10 µg/ml in PBST) for 90 min at RT and postfixed in 4% PFA for 20 min at RT. Subsequently, embryos were incubated in Equilibration Buffer (provided in ApopTag *In Situ* Apoptosis Kit) for 1h at RT. The TdT enzyme was diluted in Reaction Buffer (provided in the kit) (30%, v/v). Embryos were incubated in this TdT enzyme/reaction buffer solution overnight at 37°C. The DNA end-labeling reaction was stopped by transferring the embryos to working strength Stop buffer (provided in the kit) for 10 min at RT. After several PBST washes, embryos were incubated in blocking solution (2% NGS, 2 mg/ml BSA in PBST) for 2 h at RT. Subsequently, embryos were incubated in anti-Digoxygenin alkaline phosphatase conjugated Fab fragment (Roche) at a 1:3,000 dilution in PBST overnight at 4°C to detect digoxygenin labeled DNA fragments. BCIP/NBT (160 ng/ml in PBST) was used as a chromogenic substrate for alkaline phosphatase. Staining reaction was stopped by several washes in Stop solution (PBST/EDTA, pH 5.5) and embryos were transferred to 100% glycerol for clearing and storing.

### Microscopy and Image Analysis

Transmitted light images were recorded using an Axioskop compound microscope (Carl Zeiss) with DIC optics and an AxioCam MRc digital camera (Carl Zeiss). For quantification of cells at single cell resolution, images of stained embryos were acquired using an Axio Examiner D.1 (Carl Zeiss) with DIC optics and an LD LCI Plan Apochromat 20× (NA = 1.0) objective. Z-stacks were recorded in 2 µm steps using the ZEN 2010 software (Carl Zeiss). We used the ZEN 2010 Blue software to count cell numbers in z-stacks. The image stack was converted to greyscale to enhance contrast. We used the ZEN 2010 Blue Event Marker tool to manually label each cell in the x, y, and z plane. We validated that each cell was counted only once by re-tracking each cell through the z-stack. For images representing distributions of neurons in z-stacks, minimum intensity projections were generated in ImageJ or ZEN 2010 Blue.

### Statistics

In the primary screen, all compounds except OF-1 and AGK-1 were tested at 1, 3, 10, 30 µM with 7 to 16 embryos in each treatment (no independent replicates; **Supplementary Table 1** report data for compounds with phenotype, **Supplementary Table 3** reports compounds without phenotype). All compounds including OF-1 and AGK-1, but excluding PFI-2 and UNC1215, were also tested at 100 µM with at least 10 embryos in each treatment (two independent replicates; **Supplementary Table 2**). For all 10 compounds that generated changes in *th* expression, one additional experiment was performed, such that data were obtained at least in duplicate (**Supplementary Table 1**). In the secondary screen, all *th* WISH assays with cell counts were performed at least in triplicates (three to six independent experiments; **Supplementary Table 4**). The *isl1* assays with cell counts were performed in triplicate (**Supplementary Table 5**). The quantitative evaluation of *sox2* expression was performed in triplicate (**Supplementary Table 6**). The apoptosis assays with cell counts were performed in triplicates (**Supplementary Table 7**), except for Vorinostat, Mocetinostat, and JQ1, which were only performed in duplicate. Retina diameters of inhibitor and DMSO treated embryos were determined in three to six replicates (**Supplementary Table 8**). Retina diameters of 72 and 120 hpf control embryos were determined in a single experiment on eight embryos each.

Statistical analyses were performed using GraphPad Prism 6 software (GraphPad Software). Embryos from the WISH experiments were scored according to staining intensity and distribution. Comparison of phenotypes of DMSO treated control embryos and small molecule compound treated embryos was analyzed by two-way Fisher's Exact Test. Cell numbers and retina diameter measurements from independent experimental replicates were analyzed using two-way-ANOVA with *ad hoc* Bonferroni correction for multiple comparisons. For retina diameter comparison to 72 and 120 hpf larvae, statistical analysis used Mann-Whitney test. Graphs show mean cell numbers with standard deviation. For cell counts or numbers of embryos affected by a given phenotype, the averages for control embryos were set to 100 (phenotypes) or 1.0 (cell numbers), and all measurements of treated embryos were normalized with respect to the control value. **Supplementary Tables 4****–****8** provide the detailed statistical analysis for each experiment.

## Results

### A Chemical Genetics Screen Broadly Targeting Epigenetic Mechanisms

**Figure 1:**
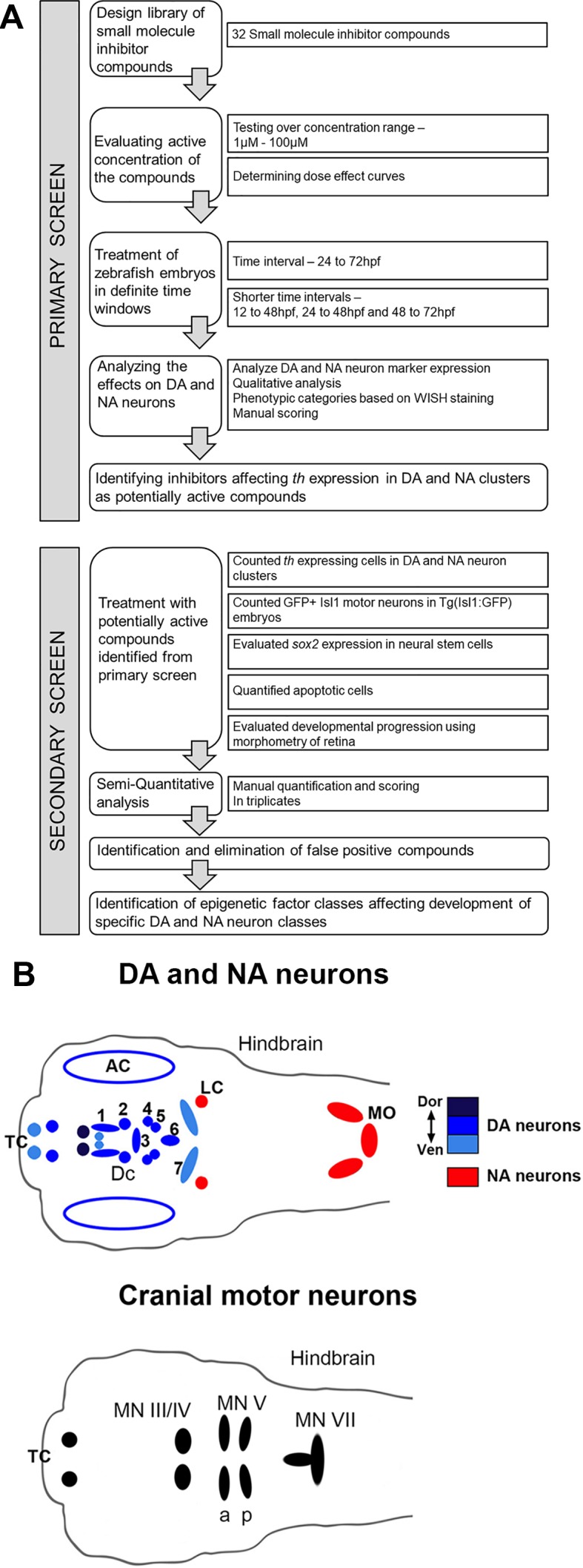
Design of Small Molecule Screen in two phases. **(A)** Schematic depiction of primary screening and secondary screening procedure. We selected 32 small molecule inhibitors that target chromatin regulators. Zebrafish larvae were exposed to the small molecule inhibitors during distinct phases of DA and NA neuron development ranging from 24 to 72 hpf. We screened all compounds at different concentration to find the optimal dosage for each small molecule inhibitor. During the primary screen we evaluated the effects of all selected small molecule inhibitors on DA and NA neurons by whole mount *in situ* hybridization (WISH) analysis of *tyrosine hydroxylase* expression. Small molecules that caused a dose-dependent change in DA and NA marker expression were considered a screening hit. For these compounds, we performed a secondary screen to evaluate DA and NA neurogenesis steps affected. **(B)** Schematic drawings of the distribution of DA and NA neurons (top, taken from Mahler et al., 2010), as well as of Isl1 expressing cranial motor neurons in the larval zebrafish brain at 96 hpf. DA neurons (blue, color intensity encodes dorsal to ventral position of DA groups as indicated) analyzed during the screening procedure reside in the olfactory bulb, subpallium (telencephalon, Tc), retina, pretectum (PrT) and ventral diencephalon (DC1–6) (Holzschuh et al., 2001; Rink and Wullimann, 2002). NA neurons (red) reside within the locus coeruleus (LC) and medulla oblongata (MO) area of the hindbrain. The analyzed Isl1 expressing cranial motor neurons reside within the telencephalon (Tc), the midbrain (MN II/IV) and the hindbrain (MN Va, Vp and MN VII) (Higashijima et al., 2000). AC, amacrine cells; DA, dopaminergic; DC, diencephalic; LC, locus coeruleus; MN, motor neuron cluster; MO, medulla oblongata; NA, noradrenergic; Tc, telencephalon.

To identify chromatin regulatory mechanisms that function during embryonic DA neurogenesis *in vivo*, we performed a chemical genetics screen in zebrafish embryos (**Figure 1A**). The screen was organized in two phases: in the primary screen, we tested 32 compounds to establish lethal concentrations and identify compounds with effects on DA neurogenesis based on changes in *th* expression. In the secondary screen, we compared effects on DA neurons with other neuronal population and tried to distinguish effects on NSCs, neuronal differentiation and survival.

**Figure 2:**
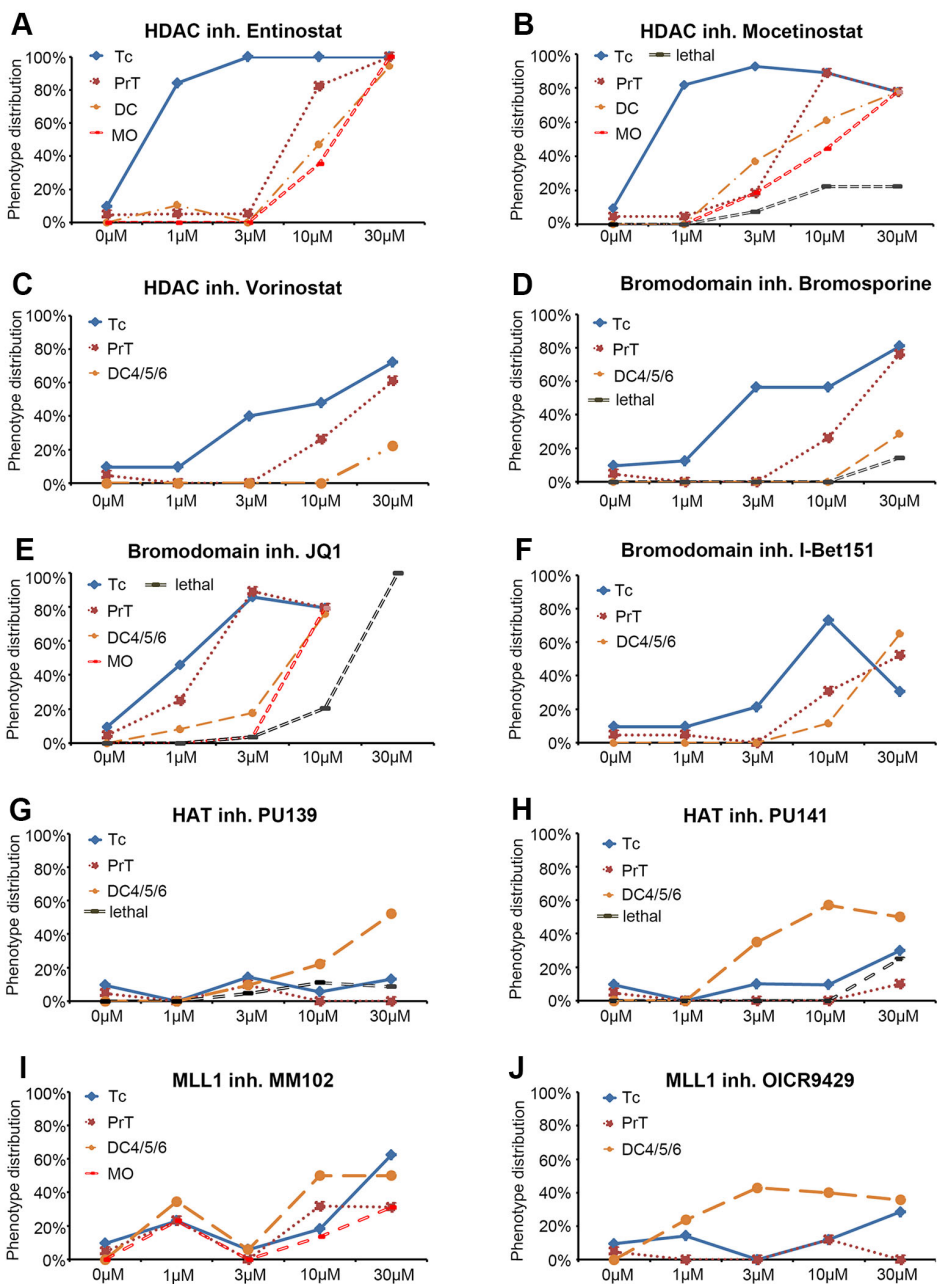
Dose response curves for selected small molecule inhibitors. **(A–J)** Dose response curves for HDAC inhibitors **(A)** Entinostat, **(B)** Mocetinostat, **(C)** Vorinostat, for Bromodomain inhibitors **(D)** Bromosporine, **(E)** JQ1, **(F)** I Bet151, for HAT inhibitors **(G)** PU139, **(H)** PU141, and for MLL1-WDR5 interaction inhibitors **(I)** MM102 and **(J)** OICR9429. Each line represents a specific DA or NA neuron cluster for which a change in *th* expression was observed. The black dashed line (“lethal”) indicates the percentage of embryos that died at a given compound concentration. Each data point represents the percentage of embryos showing an effect on the specific neuron cluster at the depicted concentration. Abbreviations for dopaminergic clusters: Tc, telencephalon; PrT, pretectum; DC, diencephalic clusters 1–6; noradrenergic clusters; MO, medulla oblongata.

We reasoned that applying active compounds during specific stages of DA neuron development might affect chromatin regulation in NSCs, DA progenitor populations, or mature neurons. Based on previous reported cell permeability and *in vivo* activity, we selected 32 small molecule compounds that target different groups of chromatin regulators (**Supplementary Figure 1**). This set includes 6 histone deacetylase (HDAC) inhibitors, two sirtuin inhibitors, 4 histone acetyltransferase (HAT) inhibitors, 8 bromodomain inhibitors, 7 histone lysine methyltransferase (HKMT) inhibitors (including 2 MLL1-WDR5 interaction inhibitors), 4 histone lysine demethylase (HKDM) inhibitors, and 1 histone methyl reader protein inhibitor (**Supplementary Figure 1**). The active working concentrations and lethal concentrations for these compounds were mostly unknown for zebrafish. We tested each compound at 1, 3, 10, and 30 µM concentrations to determine if any compound showed lethal effects in the postulated range of active concentration (see black interrupted lines in **Figure 2**; lethality data for all compounds are reported in **Supplementary Tables 1****–****3** ). Previous small molecule screens in zebrafish embryos reported high hit rates and low off-target toxicity within this dose range (Peterson and Fishman, 2011). We then evaluated the dose–response curves for each of these compounds with regard to DA or NA neurogenesis. Small molecule inhibitors that caused no overall morphological alterations were subsequently also tested at 100 µM concentration, which however, in many cases turned out to be lethal (**Supplementary Table 2**).

**Figure 3:**
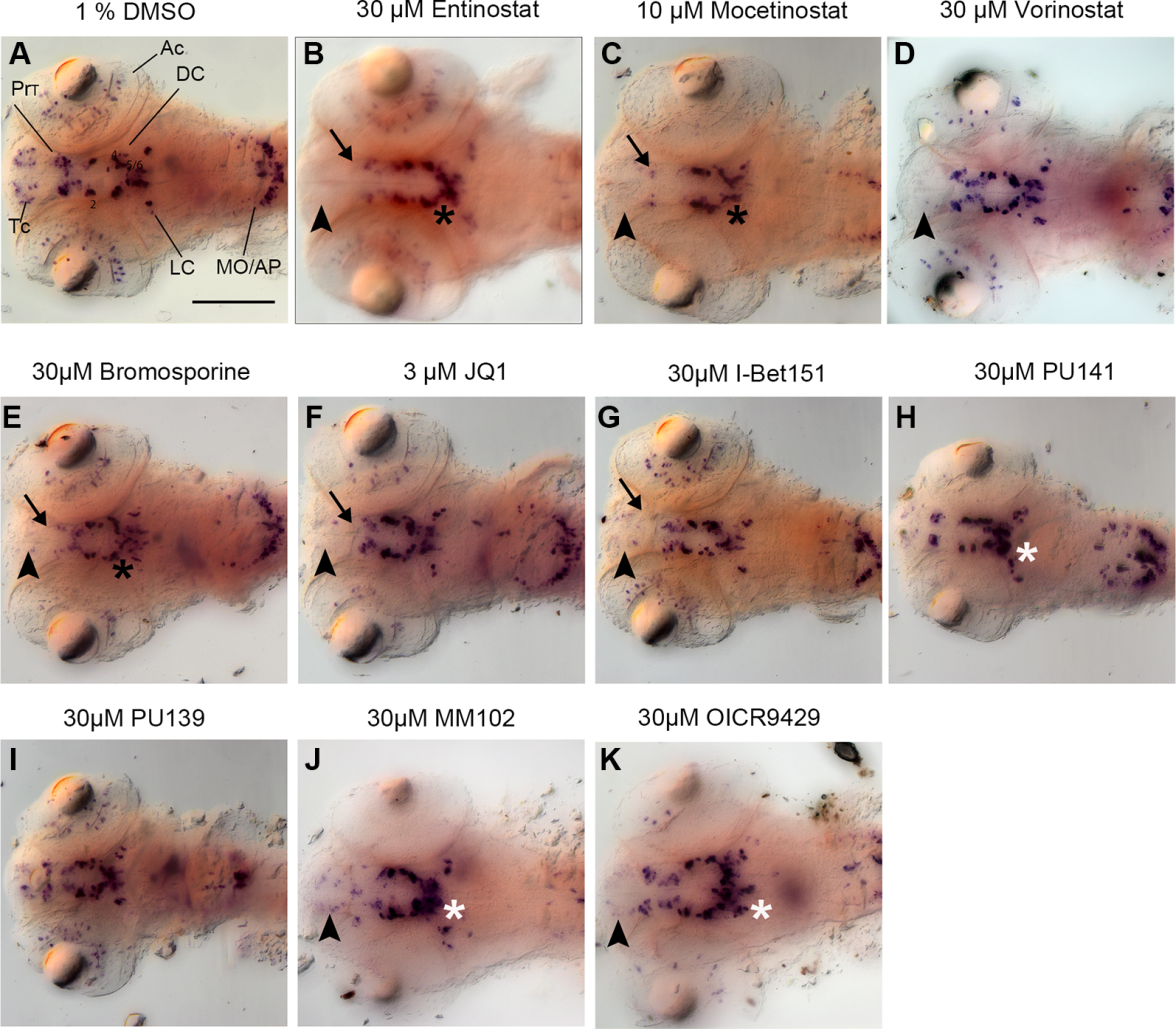
Primary Screen results for representative small molecule inhibitors. **(A–K)** Expression of the DA and NA neuronal marker *th* detected by whole mount *in situ* hybridization in embryos fixed after drug or control treatment at 96 hpf. Treatments are indicated above each panel. During the primary screen *th* expression was analyzed in DA clusters in telencephalon, pretectum, and diencephalic groups 1–6. Dorsal views of heads of larvae, images generated from Z-Projections of image stacks. Neuronal groups with reduced WISH stain are indicated as follows: Black arrowheads for telencephalic DA clusters **(B–G, J, K)**, black arrows for pretectal DC clusters **(B, C, E–G)**, black asterisks for DC DA clusters **(B, C, E)**. White asterisks indicate increased stain intensities in DC DA clusters **(H–K)**. Scale bar in **(A)** is 100 µm for all panels. AC, amacrine cells; Tc, telencephalon; PrT, pretectum; DC, diencephalic groups; Lc, locus coeruleus; MO/AP, medulla oblongata/area postrema.

Zebrafish DA and NA neurons develop in temporally distinct phases of neurogenesis (Mahler et al., 2010). We exposed zebrafish embryos to small molecule compounds during a broad interval of DA and NA neuron development, while aiming not to interfere with early pattern formation in embryogenesis. Therefore, in our primary screen, we added the compounds at 24 hpf and incubated embryos until 72 hpf. After removal of the compounds, embryos developed for another 24 h in standard E3 medium until fixation at 96 hpf for further analyses. All incubation media also contained PTU to suppress pigmentation (Elsalini and Rohr, 2003) and facilitate imaging of the brains. Treated embryos were assayed for DA and NA neurogenesis defects at 96 hpf by WISH using *th* expression as a marker for DA and NA neurons (Holzschuh et al., 2003). The *th* expressing DA clusters in the telencephalon (Tc), the pretectum (PrT), and the diencephalon (DC1–DC6) were assayed individually. We determined olfactory bulb and subpallial DA groups, which undergo neurogenesis in similar time windows (Mahler et al., 2010), together as telencephalic DA groups. The locus coeruleus (LC) NA cluster was analyzed separately, while NA clusters within the medulla oblongata (MO) and area prostrema (AP) were analyzed together and labeled MO/AP. The DA and NA phenotypes were classified into three categories based on the level and pattern of the *th* transcript detection: (1) no differences detected compared to the DMSO treated controls, (2) increase or (3) decrease in cell numbers and/or stain intensity. While we refined the analysis by cell counts in the secondary screen, in the primary screen we did not distinguish increase in cell number or *th* WISH stain intensity. Dose-effect curves were calculated and display the percentage of treated embryos showing altered *th* staining intensity for each compound concentration (**Figure 2**). **Figure 3** shows only compounds that affected DA or NA development in treated embryos. For examples of compounds with no effects on *th* expression see **Supplementary Figure 2**. We considered those small molecule compounds that caused a change in *th* mRNA expression intensity (compared to DMSO controls) in more than one third of tested embryos as a compound potentially affecting DA and NA neurogenesis, and selected them for further characterization in the secondary screen (**Figures 1A, B**).

### Compounds Identified in the Primary Screen

**Figure 4:**
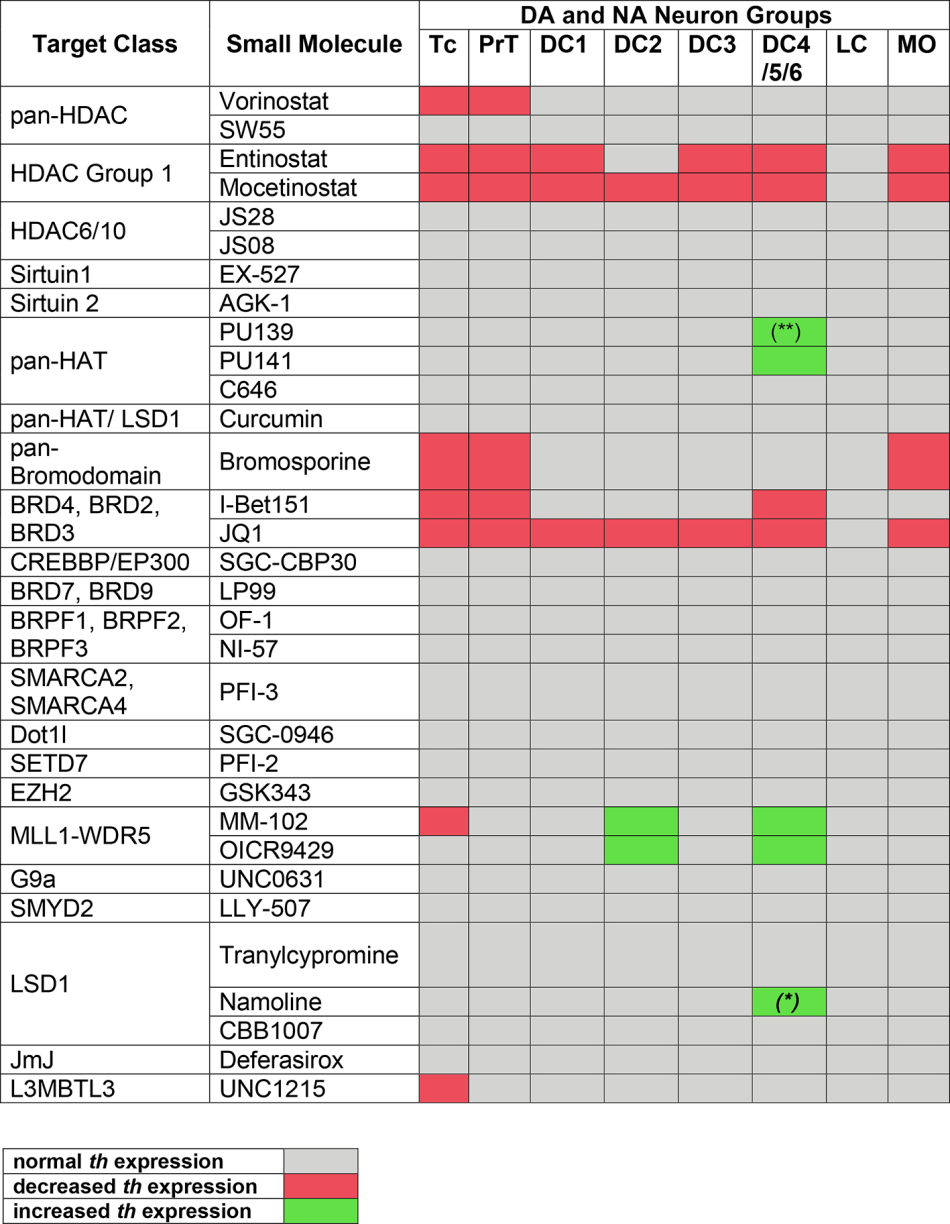
Summary of Primary Screening Results. Table depicting effects on *th* expression observed in zebrafish embryos after treatments with inhibitors shown at left. Only when treatment with at least one compound concentration caused one third or more of the assayed embryos to be affected, *th* expression was classified as decreased or increased, respectively. Treatments at 1 to 30 µM were considered, while 100 µM was excluded due to high lethality with most compounds. Color code at bottom: decreased or increased *th* expression represents both potentially changed expression levels or changed number of expressing cells, which could not be distinguished during qualitative analysis of images. (*) For Namoline, the increase of DC4/5/6 was only observed at 10 µM. (**) For PU139, only 7 out of 23 embryos showed a slight increase in stain intensity.

Among 32 compounds tested, the primary screening identified 10 compounds to affect *th* expression in the larval brain (**Figures 2**, **3**, **4**; **Supplementary Table 1**). Namoline caused changes only at one concentration, and UNC1215 had effects that did not appear to be dose dependent. Therefore, we did not further analyze these compounds. Treatments with the other 20 compounds resulted in normal *th* expression within the brain of more than two thirds of treated embryos (**Supplementary Figure 2**; **Supplementary Table 3**). We found that some compounds interfering with histone acetylation and acetylation reader proteins containing Bromodomains decrease *th* expression in treated embryos. The HDAC class 1 inhibitors Entinostat and Mocetinostat reduced *th* expression within telencephalic, pretectal and diencephalic DC1 DA groups in zebrafish larvae (**Figures 2A, B**, **3B, C**, and **4**). We also observed a less pronounced decrease within the DC4/5 and 6 DA groups and MO/AP NA groups (**Figures 2A, B**). The observed alterations in *th* expression in Tc, PrT and DC were dose-dependent, with higher concentrations affecting higher percentages of treated embryos (**Figures 2A, B**). The concentration-dependent effects became prominent at concentrations of 10 µM and higher. More than 80% of treated embryos showed altered *th* expression in Tc, PrT and DC groups in treatments with 10 or 30 µM of both compounds (**Figures 2A, B**). The pan-HDAC inhibitor Vorinostat (SAHA) affected *th* expression in Tc and PrT DA groups (**Figures 2C**, **3D**, and **4**). The effects on *th* expression in Tc and PrT were dose-dependent with more than 60% of treated embryos showing reduced *th* expression at 30 µM.

We observed a reduction in *th* expression upon treatment with the pan-Bromodomain inhibitor Bromosporine and the Bet-Bromodomain inhibitors I-Bet151 and JQ1 (**Figures 2D–F**, **3E–G**, and **4**). Treatments with Bromosporine, in a dose-dependent manner, caused reduced *th* expression within the Tc and the PrT DA groups and the MO/AP NA groups (80% at 30 µM; **Figures 2D** and **3E**). Furthermore, we detected a minor reduction in *th* staining intensity in DC4/5/6 groups in 20% of treated embryos at 30 µM (**Figure 2D**). Treatments with JQ1 reduced *th* expression in Tc, PrT, and DC4/5/6 DC groups and the MO/AP NA groups at 10 µM (80% of treated embryos; **Figures 2E**, **3F**, and **4**). I-Bet151 also caused a reduction in *th* expression within the Tc, PrT and DC4/5/6 DA groups (**Figures 2F** and **3G**).

Embryos treated with either pan-HAT inhibitor PU139 or PU141 showed slightly increased *th* stain intensity in DA groups DC4/5/6 at 30 µM (**Figures 2G, H**, **3H, J**, and **4**). Treatments with each compound at 30µM also caused morphological abnormalities as evident in a smaller head size (**Figures 3H, I**).

We assayed two compounds that specifically disrupt the interaction between histone methyltransferase MLL1 and its binding partner WDR5. For MM102 we found reduced *th* expression in Tc, but surprisingly at 10 and 30 µM *th* expression appeared increased in DC4/5/6 DA groups (**Figures 2I, J**, **3J, K**, and **4**). For PrT and DC1 DA groups, the effect was variable; with few embryos having increased or decreased *th* expression (see also **Supplementary Table 2**). We observed similar effects with the MLL1-WDR5 interaction inhibitor OICR9429 (**Figures 2J**, **3K**, and **4**). At compound concentrations between 3 and 30 µM we found 30% of treated embryos to show increased *th* staining intensities in DC4/5/6 (**Figures 2J** and **3K**).

Distinct DA and NA groups develop in distinct temporal waves of neurogenesis during development (Mahler et al., 2010). To investigate whether specific DA and NA neuron progenitor pools may be sensitive to epigenetic inhibitors in shorter time windows of neurogenesis, we applied HDAC, HAT, bromodomain and MLL1 interaction inhibitors for 24 or 36 h time windows (12 to 48 hpf, 24 to 48 hpf, or 48 to 72 hpf). These treatments resulted in similar decreases in *th* expression in Tc and PrT DA neurons for each time window (**Supplementary Figure 3**). However, early born DC2 DA neurons were differently affected in specific time windows by Entinostat and Mocetinostat: the effect of early treatment starting at 12 hpf on DC2 DA neurons was stronger when compared to treatments starting at 24 hpf or later, suggesting that these HDAC inhibitors might act on DC2 DA progenitor cells. We conclude that different phases of neurogenesis are differentially affected by these HDAC inhibitors.

### Secondary Screen Assessing Additional Neuron Types, Neural Stem Cells, and Cell Death

In a secondary screen, we (1) quantified the effects of the compounds on DA and NA cell numbers, and tested whether (2) compound effects are selective for DA or NA populations or also affect other neuronal types, (3) effects on DA or NA neurons may be from loss of neural stem cells, (4) compounds may cause apoptosis, and (5) compounds may interfere with normal developmental progression and morphogenesis (**Figure 1B**). We screened in detail the 10 inhibitors active in our primary screen: We chose a 30 µM concentration, except for Mocetinostat (10 µM) and JQ1 (3 µM), based on our primary screen results. Compounds were applied from 24 until 72 hpf, and embryos analyzed at 96 hpf.

(1) To independently validate and quantify compound effects on DA or NA neuron number, we repeated the WISH analysis for *th* on treated embryos at 96 hpf. We recorded high-resolution z-stacks of the brains from control and treated embryos, and counted *th* expressing cells in specific DA and NA cell clusters. We also compared subcellular localization of the *th* WISH signal in pretectal DA and locus coeruleus NA neurons, which were imaged at highest resolution, and found *th* transcript to be localized predominantly in the cytoplasmic compartment for DMSO controls and compound treated embryos. (2) Given that chromatin regulation mechanisms may not be selective for DA or NA neurons, we analyzed whether the compounds also affect other types of early developing neurons. To visualize *islet1* expressing neurons, which include primary and secondary cranial and spinal motor neurons, but also other neuron populations in the forebrain, we used transgenic Tg(*isl1:GFP*)^rw0/+^ zebrafish (Higashijima et al., 2000) and stained 96 hpf embryos by anti-GFP immunohistochemistry. The DA or NA neuronal groups and the Isl1:GFP expressing neuronal groups are anatomically distinct, except for the prethalamic DC1 DA cluster, which requires *isl1* for differentiation of DA neurons (Filippi et al., 2012). In our analysis of Isl1:GFP transgene expressing cranial motor neurons we did not include the Isl1 positive DC1 DA neurons. *islet1* positive neurons were documented in image stacks and selected neuron groups were counted. (3) NSC states, including self-maintenance and segregation of progenitors, rely on chromatin regulation and may be affected by epigenetic inhibitors. Therefore, we analyzed *sox2* expression, a stem cell marker expressed in all NCSs in the ventricular zone, by WISH of treated embryos fixed at 96 hpf, documented them, and visually evaluated effects of inhibitor exposure semi-quantitatively based on *sox2* staining intensity. (4) We further asked whether a reduction in cell numbers may be due to increased apoptosis in inhibitor treated embryos. Previously, several small molecule inhibitors targeting HDACs and Bromodomain proteins have been reported to trigger apoptotic cell death in different cancer cell lines (Mertz et al., 2011). Therefore, we performed TUNEL assays on treated zebrafish embryos fixed at 96 hpf and quantified TUNEL positive cells in defined brain regions (nasal placode NP, telencephalon Tc, retina, and hindbrain HB; diencephalon and midbrain DC/MB were counted together because the boundary could not be distinguished in dorsal views) by cell counting. (5) Another possible reason for the reduction in cell numbers could be that the compounds slow down development of the larvae, resulting in a developmental delay as compared to the DMSO treated control larvae. To account for any such effects, we analyzed the zebrafish retina by morphometry. We used fixed 96 hpf larvae from the *th* WISH analysis. We measured the diameter of the right and left retinae at their maximum rostro-caudal extension in inhibitor treated larvae and compared them with the age-matched DMSO treated control larvae. To determine the potential extent of delay, we also measured retinae in DMSO treated control larvae at 72, 96, and 120 hpf.

We performed all five assays in at least three independent experimental replicates. For better comparison, cell counts were normalized to the cell counts obtained from DMSO treated control embryos. To assess whether differences between control and inhibitor treated embryos may be significant, we performed two-way ANOVA.

### MLL1-WDR5 Interaction Inhibitors MM102 and OICR9429

In the primary screen, the MLL1-WDR5 interaction inhibitors MM102 and OICR9429 emerged as potential hits. However, the qualitative primary analysis was not confirmed in the secondary screen: we observed that both OICR9429 and MM102 did not produce any significant changes in the number of *th* expressing cells in any of the DA or NA clusters (**Supplementary Figures 4A** and **5A–C**). We also found no effects of MM102 on the number of *isl1:GFP* transgene expressing cells and *sox2* expressing cells in the ventricular zone (**Supplementary Figures 4J, O** and **5F, I**). OICR9429 induces a small increase in the *isl1:GFP* transgene expressing cells in the midbrain clusters MNIII/IV and hindbrain cluster MNVII but no specific effects were observed on the *sox2* expressing cells (**Supplementary Figures 5D–I**). We did not observe any significant effects on apoptosis in OICR9429 or MM102 treated embryos (**Supplementary Figures 4T and 5J–L**). The morphometric analysis of retinae did not reveal delayed development in OICR9429 or MM102 treated larvae (**Supplementary Figure 6A**). With respect to *th* expression, MM102 and OICR9429 appear to have been false positive hits in the primary screen. However, at least for OICR9429, the *isl1:GFP* phenotype reveals some compound activity. A potential explanation for the differences in qualitative versus quantitative *th* expression analysis may be that the cell clusters may have appeared slightly more dispersed, while the cell numbers in each cluster were actually not affected.

### HDAC Class 1 Inhibitors Entinostat and Mocetinostat

**Figure 5:**
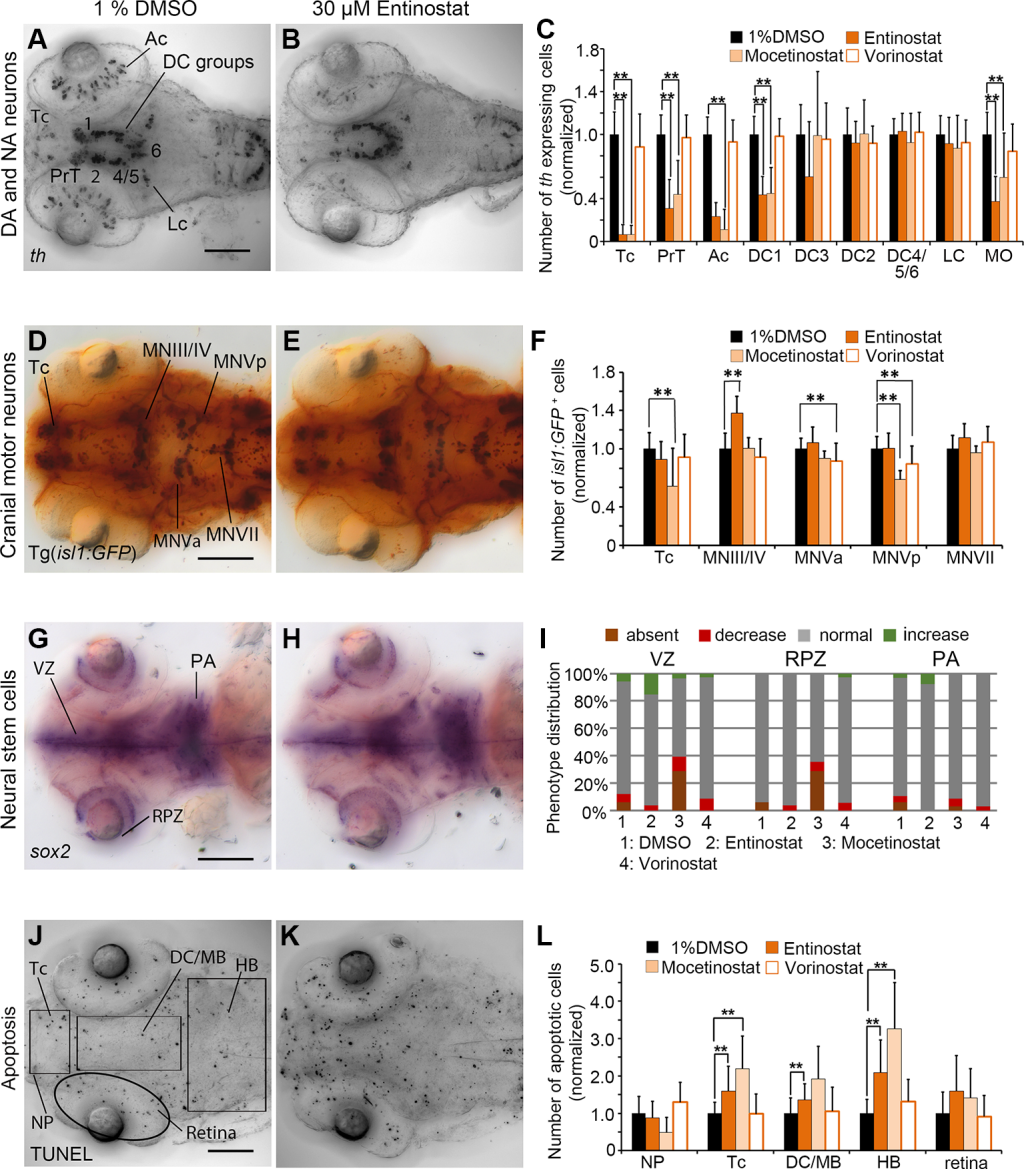
Quantification of Secondary Screen—HDAC inhibitors. **(A–C)** Analysis of *th* expression (WISH) for DA and NA neuron development. **(D–F)** Immunohistochemistry for Isl1-GFP positive cranial motor neurons. **(G–H)** WISH analysis of *sox2* expression in neural stem cells. **(J–L)** TUNEL assay to detect apoptotic cells. Embryos were treated with the HDAC inhibitors Entinostat, Mocetinostat or Vorinostatat from 24 to 72 hpf and fixed at 96 hpf. **(A, B, D, E, G, H, J, K)** Dorsal views of heads of larvae, images generated from Z-Projections of image stacks, anterior at left. Scale bars represent 100 µm. Bar charts illustrate the mean cell count numbers of each neuronal subtype for **(C)** *th* expressing cells, **(F)** *isl1:GFP* transgene expressing cells, **(L)** apoptotic cells. Error bars depict standard deviations of the means. Asterisks indicate significant differences compared with the 1% DMSO control (p < 0.001). **(I)** For analysis of *sox2* expression, embryos were classified into absent, decreased, normal or increased *sox2* expression phenotypes (see color code) and embryo numbers were normalized to 100%. AC, amacrine cells; DA, dopaminergic; NA, noradrenergic; Tc, telencephalon; PrT, pretectum; DC, diencephalic groups; Lc, locus coeruleus; MO/AP, medulla oblongata/area postrema; MN, motor neuron cluster; VZ, ventricular zone; RPZ, retinal proliferation zone; PA, pharyngeal arches; NP, nasal placodes; DC/MB, diencephalon and midbrain region; HB, hindbrain.

In Entinostat and Mocetinostat treated embryos, *th* WISH cell counts confirmed the phenotypes observed in the primary screen, while Vorinostat treated embryos with respect to DA and NA cell numbers did not differ significantly from controls. For Entinostat and Mocetinostat we counted significantly less *th* expressing cells within Tc, PrT, retina and the diencephalic cluster DC1 (p < 0.001; **Figures 5A–C**; **Supplementary Figure 4B**). In addition, we also found a decrease in hindbrain NA medulla oblongata/area postrema neurons (MO/AP; p < 0.001; **Figures 5A–C**). We could not detect any changes in cell numbers in DA neuron group DC2 and NA neurons in the locus coeruleus (LC). Both neuron populations become postmitotic before the onset of the small molecule compound exposure (Mahler et al., 2010), suggesting that Entinostat and Mocetinostat do not affect the maintenance of differentiated DA and NA neurons. These HDAC inhibitors also influence cell numbers of *isl1* positive neurons (**Figures 5D–F**; **Supplementary Figure 4G**). Entinostat treatments resulted in an increase in cell number only within the midbrain motor neuron clusters MNIII/IV (**Figures 5D–F**; p < 0.001). However, Mocetinostat treatments caused a reduction in *isl1* positive neurons in Tc (p < 0.001), and hindbrain motor neuron cluster MNVp (p < 0.001) (**Figure 5F** and **Supplementary Figure 4G**). Vorinostat affects *isl1* positive neurons in MNVa and MNVp (**Figure 5F**). *Sox2* expression in NSCs within the ventricular zone (VZ) and the retina proliferation zone was unaffected in Entinostat and Vorinostat treated embryos (**Figures 5G–I**). However, Mocetinostat strongly reduced *sox2* expression exclusively in the ventricular zone and the retina proliferative zone (**Figure 5I**; **Supplementary Figures 4K, L**). We did not observe a reduction of *sox2* transcript within the pharyngeal arches, indicating that *sox2* expression in NSCs may be selectively affected. Treatments with Entinostat and Mocetinostat caused an overall increase in apoptosis in the zebrafish brain based on TUNEL assays. We counted significantly more apoptotic cells in the telencephalon, di-/mesencephalic area and hindbrain in Entinostat treated zebrafish larvae (**Figures 5J–L**; p < 0.001), and in the telencephalon and hindbrain of Mocetinostat treated larvae (p < 0.001, see also **Supplementary Figure 4Q**). Vorinostat did not affect apoptosis (**Figure 5L**). The morphometric analysis of the retina diameter showed no significant changes in the retinae in Vorinostat and Mocetinostat treated larvae compared to the 96 hpf DMSO treated control larvae, indicating no developmental delay. In case of Entinostat treated larvae, the mean diameter of the retinae showed an increase of 10.65% (p < 0.001), which is higher than the change in mean diameter during normal developmental progression from 96 to 120 hpf (**Supplementary Figure 6**), arguing for hyper-proliferation or morphological expansion of the retina.

### pan-Bromodomain and Bet-Bromodomain Inhibitors

**Figure 6:**
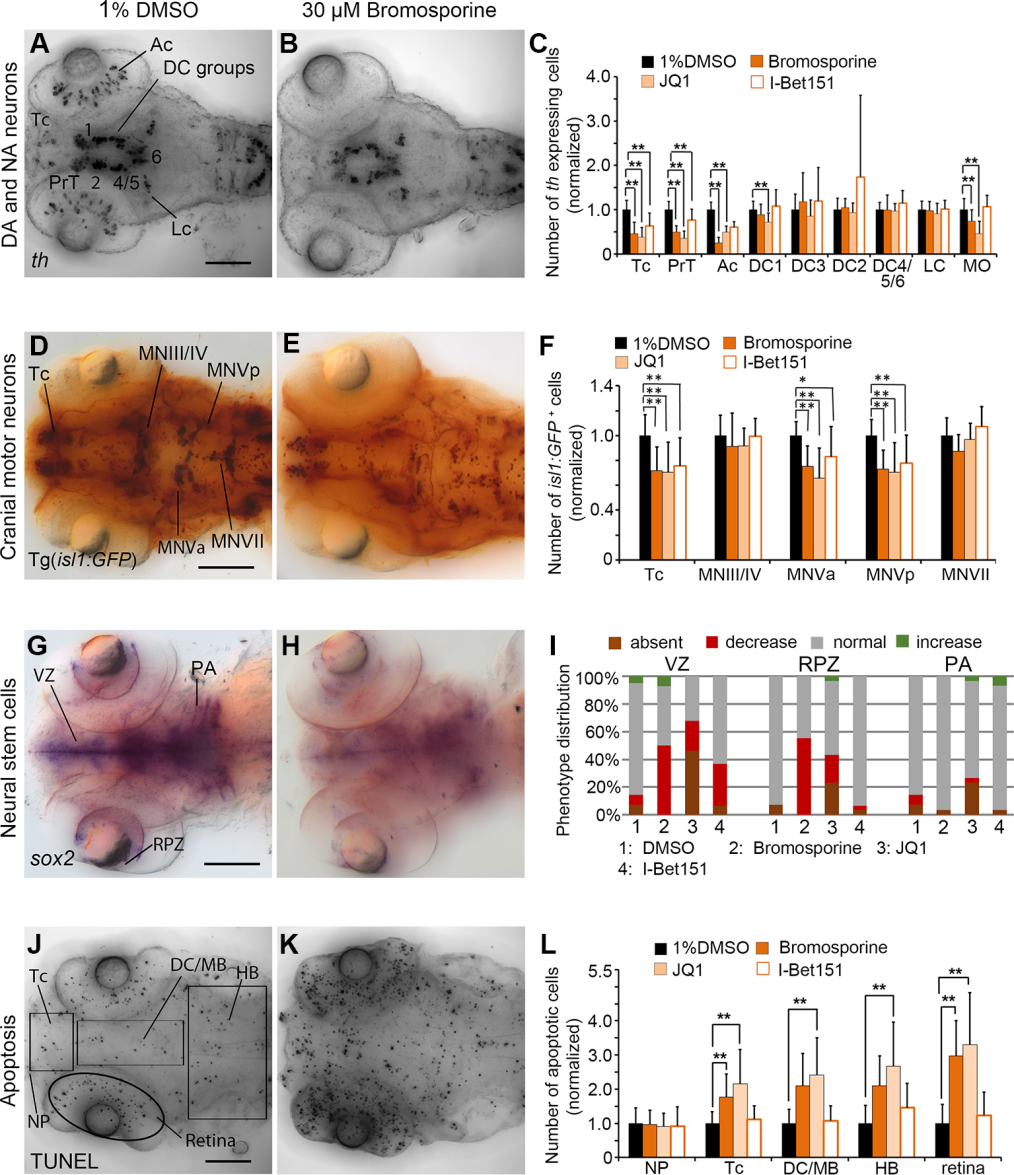
Quantification of Secondary Screen—Bromodomain inhibitors. **(A–C)** Analysis of *th* expression (WISH) for DA and NA neuron development. **(D–F)** Immunohistochemistry for Isl1-GFP positive cranial motor neurons. **(G–I)** WISH analysis of *sox2* expression in neural stem cells. **(J–L)** TUNEL assay to detect apoptotic cells. Embryos were treated with the Bromodomain inhibitors Bromosporine, JQ1 or I-Bet151 from 24 to 72 hpf and fixed at 96 hpf. **(A, B, D, E, G, H, J, K)** Dorsal views of heads of larvae, images generated from Z-Projections of image stacks, anterior at left. Scale bars represent 100 µm. Bar charts illustrate the mean cell count numbers of each neuronal subtype for **(C)** *th* expressing cells, **(F)** *isl1:GFP* transgene expressing cells, **(L)** apoptotic cells. Error bars depict standard deviation of the mean. Asterisks indicate significant differences compared with the 1% DMSO control (p < 0.001). **(I)** For analysis of *sox2* expression, embryos were classified into absent, decreased, normal or increased *sox2* expression phenotypes (see color code), embryo numbers were normalized to 100%. AC, amacrine cells; DA, dopaminergic; NA, noradrenergic; Tc, telencephalon; PrT, pretectum; DC, diencephalic groups; Lc, locus coeruleus; MO/AP, medulla oblongata/area postrema; MN, motor neuron cluster; VZ, ventricular zone; RPZ, retinal proliferation zone; PA, pharyngeal arches; NP, nasal placodes; DC/MB, diencephalon and midbrain region; HB, hindbrain.

The pan-Bromodomain inhibitor Bromosporine as well as the Bet-Bromodomain inhibitors JQ1 and I-Bet151 caused a severe reduction in *th* expression within DA clusters in TC and PrT in the primary screen. Embryos treated with Bromosporine or I-Bet151at 30 µM or JQ1 at 3 µM had reduced numbers of *th* expressing cells within TC and PrT (p < 0.001; **Figures 6A–C**; **Supplementary Figure 4C**). Furthermore, we detected less *th* expressing neurons in the retina after treatments with Bromosporine and JQ1 (p < 0.001; **Figures 6A–C**). Cell numbers of *th* expressing NA neurons in the MO/AP cluster in the hindbrain were reduced after treatments with Bromosporine and JQ1 but were unaffected by I-Bet151 treatments. These effects were not restricted to DA or NA neurons, since in bromosporine treated embryos *isl1* neurons were also reduced in the Tc and the hindbrain cluster MNV (p < 0.001; **Figures 6D–F**). We observed a similar reduction in *isl1* neurons in Tc and MNV in embryos treated with JQ1 and I-Bet151 (p < 0.001 each; **Supplementary Figure 4H**). Bromosporine caused a strong decrease in *sox2* expression in NSCs in the ventricular zone and retinal proliferation zone (p < 0.001; **Figures 6G–I**), while pharyngeal s*ox2* expression was unaffected. JQ1 also causes a strong decrease in *sox2* expression in NSC in the VZ and RPZ (**Figure 6I**). Loss of *sox2* expression in NSCs suggests that reduced numbers of *th* and *isl1:GFP* expressing neurons may be caused by a depletion of *sox2* expressing NSCs. I-Bet151 also resulted in reduction of *sox2* expression in VZ (**Figure 6I**; **Supplementary Figure 4M**). Treatments with Bromosporine and JQ1 also cause an overall increase in apoptosis. We counted significantly more apoptotic cells in telencephalon and retina of zebrafish embryos treated with bromosporine (**Figures 6J–L**; p < 0.001). JQ1 caused a significant increase in apoptosis in each of the anatomical regions analyzed except the nasal placode. Surprisingly, we did not observe any specific effect on apoptosis in the I-bet151 treated larvae. The morphometric analysis of the retinal diameters in Bromosporine, I-Bet151 and JQ1 treated larvae showed no significant changes with respect to the 96 hpf DMSO treated control larvae (**Supplementary Figure 6**). In summary, the severe effects of Bromosporine, JQ1 and I-Bet151 on DA or NA as well as *isl1* neuron development may be caused by loss of NSCs, increased apoptosis, but may also directly affect neuronal differentiation.

### pan-HAT Inhibitors PU139 and PU141

**Figure 7:**
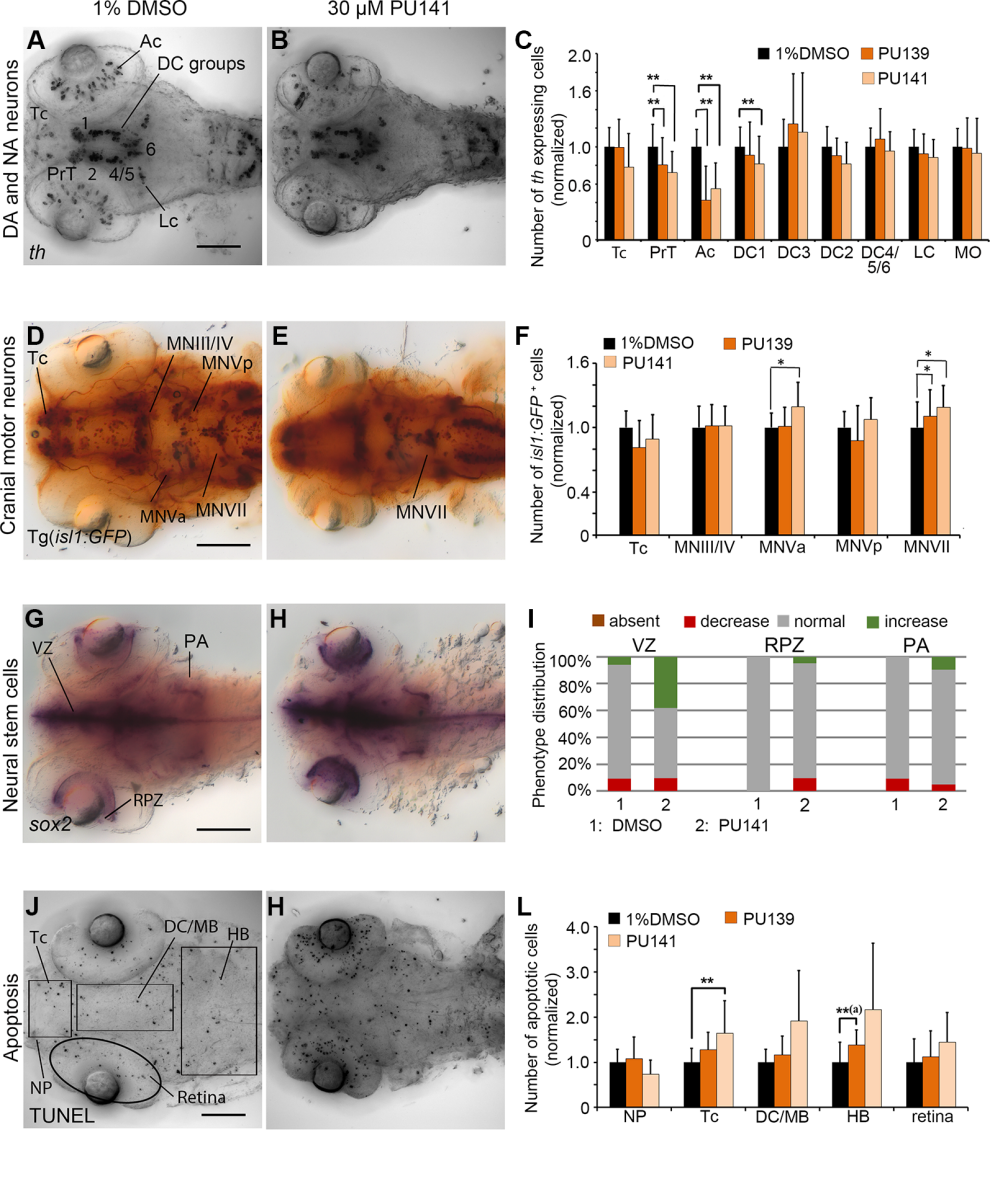
Quantification of Secondary Screen, HAT inhibitors. **(A–C)** Analysis of *th* expression (WISH) for DA and NA neuron development. **(D–F)** Immunohistochemistry for Isl1-GFP positive cranial motor neurons. **(G–I)** WISH analysis of *sox2* expression in neural stem cells. **(J–L)** TUNEL assay to detect apoptotic cells. Embryos were treated with the HAT inhibitors PU139 or PU141 from 24 to 72 hpf and fixed at 96 hpf. **(A, B, D, E, G, H, J, K)** Dorsal views of heads of larvae, images generated from Z-Projections of image stacks, anterior at left. Scale bars represent 100 µm. Bar charts illustrate the mean cell count numbers of each neuronal subtype for **(C)** *th* expressing cells, **(F)** *isl1:GFP* transgene expressing cells, **(L)** apoptotic cells. Error bars depict standard deviations of the means. Asterisks indicate significant differences compared with the 1% DMSO control (p < 0.001). **(I)** For analysis of *sox2* expression, embryos were classified into absent, decreased, normal or increased *sox2* expression phenotypes (see color code) and embryo numbers were normalized to 100%. AC, amacrine cells; DA, dopaminergic; NA, noradrenergic; Tc, telencephalon; PrT, pretectum; DC, diencephalic groups; Lc, locus coeruleus; MO/AP, medulla oblongata/area postrema; MN, motor neuron cluster; VZ, ventricular zone; RPZ, retinal proliferation zone; PA, pharyngeal arches; NP, nasal placodes; DC/MB, diencephalon and midbrain region; HB, hindbrain.

The primary screen revealed HAT inhibitors PU139 and PU141 to affect *th* expression within DA neuron groups in DC4/5/6. In contrast, cell counts revealed that PU139 or PU141 reduced the numbers of *th* expressing cells in the DA neuron clusters PrT, DC1 and in the retina (p < 0.005; **Figures 7A–C**) but not in DC4/5/6. We found *isl1* expressing MNVa and MNVII motor neurons numbers to be increased in PU141 treated embryos (p < 0.005; **Figures 7D–F**). Furthermore, *isl1:GFP* transgene expression revealed in PU141 treated embryos a failure in proper neuronal migration of motor neuron cluster MNVII, which arise in rhombomere 4 and normally subsequently migrate into rhombomere 6. Combined with the small head size of the treated embryos this observation might indicate developmental delay caused by the inhibitor. Interestingly, *sox2* expression levels appear elevated within ventricular and retinal proliferation zones after exposure to PU141 (**Figures 7G–I**), however the strength of the effect varied between treated embryos. PU141 caused a significant increase in apoptotic cells in the tel- and di/mesencephalon (**Figures 7J–L**; p < 0.001). In contrast, treatment with PU139 caused increased apoptosis in the hindbrain but no significant increase in other brain regions (**Figures 7J–L**; **Supplementary Figure 4S**; p < 0.001). The morphometric analysis of the retinal also revealed a decrease in in PU139 and PU141 treated larvae by 6.27 and 8.38% respectively (p < 0.001) as compared to the 96 hpf control larvae. This decrease is comparable to the change during normal developmental progression from 72 to 96 hpf (**Supplementary Figure 6**), suggesting that PU139 and PU141 cause severe developmental delay.

## Discussion

Epigenetic mechanisms have been difficult to link to specific developmental processes using genetic approaches, especially when studying organogenesis or neuronal differentiation, rather late events in development. This difficulty may be caused by epigenetic factors acting throughout development, with early loss-of-function phenotypes obscuring later development, or by partially redundant gene functions of families of epigenetic regulators controlling specific epigenetic mechanisms. Chemical genetic screens have been developed to address exactly these problems (Yeh and Crews, 2003), applying small molecule compounds that may affect whole classes of active proteins, and can be applied selectively during defined developmental phases. In this study, we describe a chemical genetics screening strategy to uncover chromatin regulatory mechanisms during neural development *in vivo* using zebrafish embryos. We focus our screen on DA neurons, which are well characterized in zebrafish. While midbrain DA neurons are extensively studied in mammalian systems, much less is known about development of other forebrain DA groups. However, since most forebrain DA groups are well conserved from fish to mammals, we hope that our approach may also stimulate analysis of epigenetic control of DA groups in mammalian systems.

We screened a selected set of small molecule inhibitors of canonical chromatin regulators for effects on DA neuron marker expression *in situ* in treated embryos. As a model system, zebrafish embryos are well suitable for this kind of screening strategy. Several small molecule screens in zebrafish embryos have been performed to uncover novel regulators of stem cell homeostasis, embryonic development, inflammation or behavioral states (Peterson et al., 2000; North et al., 2007; Kokel et al., 2010; Rihel et al., 2010; Robertson et al., 2014; Richter et al., 2017). In these screens, embryos or larvae are incubated in solutions of the active compounds, which are assumed to be taken up passively through skin and gills, often facilitated by including non-lethal concentrations of organic solvents like DMSO. Previous small molecule screens in zebrafish were performed by testing large numbers of compounds at preset concentrations. However, we chose to focus on small molecule inhibitors with specific activities and restricted predefined targets. We tested each small molecule to identify potentially effective and lethal concentrations and performed multiple rounds of screening to define an optimal concentration for each compound based on phenotypes and lethality. Therefore, the number of tested small molecules is limited and hit rates from our screen may not be comparable to previous large screens using zebrafish embryos. A chemical genetics approach also offers advantages to study chromatin regulator functions during development *in vivo*. Chromatin regulators are crucial for early embryonic development, and vertebrate genetic knock-out models of these proteins are often embryonic lethal (Yu et al., 1995; Schumacher et al., 1998; O'Carroll et al., 2001; Bledau et al., 2014; Andricovich et al., 2016; San et al., 2016). Furthermore, chromatin regulators often function in the context of multi-protein complexes with distinct functional subunits and cellular targets (Chen and Dent, 2014). Genetic knock-out of a single subunit might result in the disassembly of these multi-protein complexes (Dou et al., 2006). In contrast to genetic studies, small molecule inhibitors allow to study time- and dose-dependent effects of altered epigenetic states, while multi-protein complex assembly may remain unaffected. Many chromatin regulators also comprise several families of related proteins, implying functional redundancy between different members (Arrowsmith et al., 2012). Redundant functions of different protein family members might mask some phenotypes in genetic studies, but multiple genetic knock-outs in vertebrate models are often not feasible. Small molecule inhibitors often target shared structural and functional protein motifs and thus modulate the activity of several related chromatin regulators. Our *in vivo* chemical genetics approach also has limitations. Several efficacious small molecules may be missed as screening hits because of inefficient uptake by the embryo or because the compound is degraded in metabolic processes. Small molecule inhibitors targeting chromatin regulators have been developed predominantly for the treatment of cancer in humans, because many genetic alterations in human cancers lead to aberrant activity of these proteins (Marks and Breslow, 2007; Chi et al., 2010). Therefore, most small molecule inhibitors efficiently target the human orthologues of chromatin regulators and the efficacy of these compounds has been tested only in mammalian cells. Efficacy and activity studies of most of these compounds in the zebrafish model are currently missing.

In this study, we identified and further characterized seven small molecule inhibitors that affect DA neuron development in zebrafish embryos. These compounds are the HDAC class 1 inhibitors Entinostat and Mocetinostat, the pan-Bromodomain inhibitor Bromosporine, the Bet-Bromodomain inhibitors JQ1 and I-Bet151, and the pan-HAT inhibitors PU139 and PU141. Only compounds modulating histone acetylation emerged as screening hits. However, the restricted number of identified screening hits might be due to differences in bioavailability and efficacy compared to mammalian system.

In this study, we did not experimentally validate, whether the identified HDAC and Bromodomain inhibitors alter histone acetylation or recognition in zebrafish larvae. However, for most of these compounds, the specificity and activity on histone acetylation or acetylated histone recognition, respectively, have recently been reported for larval and adult zebrafish tissues (Pfefferli et al., 2014; Vleeshouwer-Neumann et al., 2015; Pinho et al., 2016; Chan et al., 2019; Sato et al., 2019). An increase in the levels of histone acetylation has been reported in zebrafish tissues after treatment with the HDAC inhibitors Entinostat, Mocetinostat and Vorinostat. Treatments of larval zebrafish from 72 until 120 hpf with Entinostat causes hyperacetylation of H3K9 as evident from Western Blot analysis of whole larval extracts as well as from acetylated H3K9 immunostain (1 µM Entinostat/MS-275; Pinho et al., 2016). Mocetinostat caused a similar hyperacetylation of histones H3 and H4 in lysates from adult zebrafish fin regenerates, as evident from Western Blot analysis (5 µM Mocetinostat/MGCD0103; Pfefferli et al., 2014). Vorinostat has been applied to zebrafish models of embryonal rhabdomyosarcoma (ERMS) and the HDAC inhibitor is able to increase the acetylation of histones H3 and H4 in zebrafish ERMS tumors (50 µM Vorinostat/SAHA; Vleeshouwer-Neumann et al., 2015). These findings indicate that the three HDAC inhibitors are bioavailable and have specific activity in zebrafish tissues. Given that we observe phenotypes in the same concentration ranges, we conclude that the phenotypes are indeed caused by changes in histone modifications. The Bet-Bromodomain inhibitors block Bet-family proteins binding to acetylated histones, and thus might inhibit active transcription (Jang et al., 2005; Loven et al., 2013). Recently, JQ1 has been reported to block active transcription and RNAP2 recruitment during zygotic genome activation in zebrafish embryos [43.8 µM at blastula stage (Chan et al., 2019); 10 µM at Blastula stage (Sato et al., 2019)]. Furthermore, live cell imaging in zebrafish embryos revealed that JQ1 efficiently abolished the binding of a bromodomain containing reporter construct to Fab-based live endogenous labeled H3K27ac sites (Sato et al., 2019). Together, these data suggest that the bromodomain inhibitor JQ1 is active in larval zebrafish tissues and specifically blocks recognition of acetylated histones. Considering the high sequence conservation of histones as well as of active sites in epigenetic factors (for example, see Pinho et al., 2016), we assume that for many epigenetic compounds active concentration range and specificity may be conserved.

We found, that HDAC, bromodomain, and HAT inhibitors had similar effects on DA neuron development. Chemical inhibition of chromatin regulators having opposing functions on chromatin, such as HDACs and HATs resulted in similar phenotypes, which may be explained by shared epigenetic mechanisms affecting both positive and negative control of DA neurogenesis. Some studies put forward that certain cell-type specific transcription factors recruit both activating and repressing factors to chromatin (Rodriguez et al., 2005). Our findings are also in concordance with recent studies that demonstrated HDAC and Bet-Bromodomain inhibitors to regulate common transcriptional networks in Myc-induced murine lymphoma cells (Bhadury et al., 2014). Embryos treated with the HAT inhibitors PU139 and PU141 appeared developmentally delayed as observed from retinal diameter and *isl1:GFP* neuron positioning. The observed embryo to embryo variation may be explained by a variable delay in the onset of DA neurogenesis in the treated embryos. A treated embryo with delayed DA marker expression would eventually be classified as a screening hit with decreased expression. General developmental delay could be caused by a broad range of mechanisms, including those affecting stem cell maintenance or proliferation. Recently, a study found that the HAT inhibitor PU141 has non-specific targets and effects in cell assays, when applied at a concentration of 10 and 100 µM (Dahlin et al., 2017). However, it was demonstrated that *in vivo* PU141 causes hypoacetylation in neuroblastoma xenograft mouse models (Gajer et al., 2015).

Our screen revealed HDACs to be involved in DA neurogenesis within specific clusters of the embryonic zebrafish brain. However, HDACs are likely not specific regulators of DA neurogenesis, since we also found *th* expressing NA neurons and *isl1:GFP* transgene expressing motor neurons affected in treated embryos. HDAC inhibitors and HDAC 1 have been previously found to be involved in neurogenesis in zebrafish. The HDAC inhibitor valproic acid negatively regulates expression of the proneural genes *ascl1a* and *ascl1b*, and leads to impaired serotonergic neurogenesis in embryonic zebrafish brains (Jacob et al., 2014). Genetic analysis of zebrafish mutants revealed that HDAC1 promotes histone acetylation in the embryonic brain to sustain core neurogenic transcriptional networks and to modulate Notch signaling activity during motor neuron generation and retinal neurogenesis (Cunliffe, 2004; Stadler et al., 2005; Harrison et al., 2011). This evidence suggests that HDACs are involved in the global regulation of neurogenesis in zebrafish.

Furthermore, inhibition of Bromodomain containing proteins interfered with DA neurogenesis as well as with *sox2* expressing stem cells in the ventricular zone. Based on the subtype specificity of the small molecule inhibitors JQ1 and I-Bet151, we presume Bet Bromodomain proteins to mediate these effects. This family contains the similar proteins Brd2, Brd3, and Brd4. They regulate transcriptional elongation in cells mediated by their interaction with PTEFB (Jang et al., 2005).The role of Bet Bromodomain proteins in development and in particular in neurogenesis remains elusive. In mice, homozygous Brd4 knock-out animals are embryonic lethal (Houzelstein et al., 2002). A recent study found that JQ1 treatments of adult mice impairs synaptic function and memory formation by blocking the transcriptional networks underlying synaptic plasticity (Korb et al., 2015). We provide evidence that Bet Bromodomain proteins might also regulate embryonic neurogenesis.

The screening data point towards a potential influence of the small molecule treatments on proliferative or early post-mitotic DA precursor cells. Previous birth date analysis revealed distinct temporal requirements for cell-cycle exit and the neurogenic switch in distinct DA precursor populations (Mahler et al., 2010). Small molecule inhibitor treatments predominantly affected development of DA cell in TC and PrT groups that show a continuous progenitor release from long-term cycling stem and precursor populations during the time interval of the chemical exposure. Compound treatments did not affect early neural plate derived neurons that differentiate during primary neurogenesis, such as DC2 DA neurons or NA neurons of the LC, which both become postmitotic prior to our chemical exposure time window. However, it was observed that HDAC inhibitors did affect the early born DC2 DA neurons when applied in an early time window beginning at 12 to 48 hpf, thus suggesting that HDAC inhibitors may be acting on the proliferation or maintenance of DA precursors. Furthermore, the Bromodomain inhibitor Bromosporine might act by a depletion of *sox2* expressing NSCs as demonstrated by a strong decrease of *sox2* expression within the ventricular zone. HDAC and Bromodomain inhibitors might act on neuronal precursors by either interfering with proliferation and self-renewal or they may affect cell-cycle exit and differentiation. To address this question, we stained for phospho-Histone 3 (pH3) and found that pH3 is only slightly reduced overall, while even enhanced in the RPZ (**Supplementary Figure 7**), suggesting that stem cell maintenance but not proliferation may be predominantly affected. Genetic studies in mice suggest that HDACs promote proliferation, since HDAC 1 mutant mice show an overall decrease in cell proliferation (Lagger et al., 2002). Zebrafish HDAC 1 mutants exhibit a similar block in proliferative precursors within the hindbrain (Cunliffe, 2004). Evidence from several cancer cell lines suggest that HDAC and Bromodomain inhibition effectively shuts down proliferation and cancer growth by targeting oncogenic gene expression profiles (Condorelli et al., 2008; Lee et al., 2015). In the zebrafish retina, HDAC 1 is required for cell-cycle exit and differentiation of mature neurons (Stadler et al., 2005). Our findings are also in line with several genetic knock-out studies that found chromatin regulators to act on the level of NSC or progenitor cell self-renewal, proliferation or differentiation capacity (Fasano et al., 2007; Lim et al., 2009; Feng et al., 2017).

Whether epigenetic mechanisms in embryonic DA neurogenesis also impact on progression in Parkinson's disease remains to be determined. Recently, there has been strong interest in epigenetic mechanisms in mammalian midbrain DA development, stem cell derived new dopaminergic neurons, as well as in potentially neuroprotective pathways that may slow down disease progression (van Heesbeen et al., 2013; Renani et al., 2019; van Heesbeen and Smidt, 2019). In animal models of Parkinson's disease, HAT inhibitors in co-treatment with L-DOPA have been suggested to have therapeutic potential (Ryu et al., 2018). However, in elderly patients, the HDAC inhibitor valproic acid has been revealed to promote parkinsonism (Mahmoud and Tampi, 2011). The genome-wide effects of epigenetic compounds may thus complicate identification of epigenetic drugs which delay disease progression with minor side effects only.

In summary, our chemical genetics screen identified chromatin modifying processes involving HDACs, HATs and Bromodomain containing proteins to participate in DA neurogenesis *in vivo*. The screen provides a first resource for the characterization of novel regulators of DA neurogenesis. Further work combining genetics and biochemical approaches are required focusing on the role of these chromatin regulators in the context of transcription factor networks to identify combined epigenetic and transcriptional control of DA neuron development.

## Data Availability Statement

All datasets generated for this study are included in the article/**Supplementary Material**.

## Ethics Statement

The animal study was reviewed and approved by Regierungspräsidium Freiburg.

## Author Contributions

MW and WD conceptualized and designed the study. MW and PS performed the experiments, assembled the figures and wrote the first draft of the manuscript. A-TH and MJ advised on the use of small molecule epigenetic inhibitors and supplied compound libraries including unpublished compounds. WD contributed to design, supervision and editing, and provided project administration and funding acquisition.

## Funding

This study was funded by the Deutsche Forschungsgemeinschaft (DFG, German Research Foundation) under Germany's Excellence Strategy—EXC-2189—Project ID: 390939984 (Gefördert durch die Deutsche Forschungsgemeinschaft (DFG) im Rahmen der Exzellenzstrategie des Bundes und der Länder—EXC-2189—Projektnummer 390939984) and the Excellence Initiative of the German Federal and State Governments (BIOSS-EXC 294). This study was supported in part by the Deutsche Forschungsgemeinschaft (DFG: GSC-4, Spemann Graduate School, and grant 322977937/GRK2344). A-TH and MJ thank the DFG for further funding within CRC992 (Medical Epigenetics).

## Conflict of Interest

The authors declare that the research was conducted in the absence of any commercial or financial relationships that could be construed as a potential conflict of interest.

## References

[B1] Abdelilah S. Mountcastle-Shah E. Harvey M. Solnica-Krezel L. Schier A. F. Stemple D. L. (1996). Mutations affecting neural survival in the zebrafish Danio rerio. Development 123, 217–227. 9007242 10.1242/dev.123.1.217

[B2] Andricovich J. Kai Y. Peng W. Foudi A. Tzatsos A. (2016). Histone demethylase KDM2B regulates lineage commitment in normal and malignant hematopoiesis. J. Clin. Invest. 126, 905–920. 10.1172/JCI84014 26808549 PMC4767361

[B3] Ang S. L. (2006). Transcriptional control of midbrain dopaminergic neuron development. Development 133, 3499–3506. 10.1242/dev.02501 16899537

[B4] Arenas E. Denham M. Villaescusa J. C. (2015). How to make a midbrain dopaminergic neuron. Development 142, 1918–1936. 10.1242/dev.097394 26015536

[B5] Arrowsmith C. H. Bountra C. Fish P. V. Lee K. Schapira M. (2012). Epigenetic protein families: a new frontier for drug discovery. Nat. Rev. Drug Discov. 11, 384–400. 10.1038/nrd3674 22498752

[B6] Barker R. A. Parmar M. Studer L. Takahashi J. (2017). Human trials of stem cell-derived dopamine neurons for parkinson's disease: dawn of a new era. Cell Stem Cell 21, 569–573. 10.1016/j.stem.2017.09.014 29100010

[B7] Bhadury J. Nilsson L. M. Muralidharan S. V. Green L. C. Li Z. Gesner E. M. (2014). BET and HDAC inhibitors induce similar genes and biological effects and synergize to kill in Myc-induced murine lymphoma. Proc. Natl. Acad. Sci. U. S. A 111, E2721–E2730. 10.1073/pnas.1406722111 24979794 PMC4084424

[B8] Bjorklund A. Dunnett S. B. (2007). Dopamine neuron systems in the brain: an update. Trends Neurosci. 30, 194–202. 10.1016/j.tins.2007.03.006 17408759

[B9] Blaess S. Ang S. L. (2015). Genetic control of midbrain dopaminergic neuron development. Wiley. Interdiscip. Rev. Dev. Biol. 4, 113–134. 10.1002/wdev.169 25565353

[B10] Bledau A. S. Schmidt K. Neumann K. Hill U. Ciotta G. Gupta A. (2014). The H3K4 methyltransferase Setd1a is first required at the epiblast stage, whereas Setd1b becomes essential after gastrulation. Development 141, 1022–1035. 10.1242/dev.098152 24550110

[B11] Cao Y. Semanchik N. Lee S. H. Somlo S. Barbano P. E. Coifman R. (2009). Chemical modifier screen identifies HDAC inhibitors as suppressors of PKD models. Proc. Natl. Acad. Sci. U. S. A 106, 21819–21824. 10.1073/pnas.0911987106 19966229 PMC2799791

[B12] Chan S. H. Tang Y. Miao L. Darwich-Codore H. Vejnar C. E. Beaudoin J. D. (2019). Brd4 and P300 confer transcriptional competency during zygotic genome activation. Dev. Cell 49, 867–881 e868. 10.1016/j.devcel.2019.05.037 31211993 PMC7201981

[B13] Chapouton P. Adolf B. Leucht C. Tannhauser B. Ryu S. Driever W. (2006). her5 expression reveals a pool of neural stem cells in the adult zebrafish midbrain. Development 133, 4293–4303. 10.1242/dev.02573 17038515

[B14] Chen T. Dent S. Y. (2014). Chromatin modifiers and remodellers: regulators of cellular differentiation. Nat. Rev. Genet. 15, 93–106. 10.1038/nrg3607 24366184 PMC3999985

[B15] Chi P. Allis C. D. Wang G. G. (2010). Covalent histone modifications–miswritten, misinterpreted and mis-erased in human cancers. Nat. Rev. Cancer 10, 457–469. 10.1038/nrc2876 20574448 PMC3262678

[B16] Condorelli F. Gnemmi I. Vallario A. Genazzani A. A. Canonico P. L. (2008). Inhibitors of histone deacetylase (HDAC) restore the p53 pathway in neuroblastoma cells. Br. J. Pharmacol. 153, 657–668. 10.1038/sj.bjp.0707608 18059320 PMC2259214

[B17] Cunliffe V. T. (2004). Histone deacetylase 1 is required to repress Notch target gene expression during zebrafish neurogenesis and to maintain the production of motoneurones in response to hedgehog signalling. Development 131, 2983–2995. 10.1242/dev.01166 15169759

[B18] Dahlin J. L. Nelson K. M. Strasser J. M. Barsyte-Lovejoy D. Szewczyk M. M. Organ S. (2017). Assay interference and off-target liabilities of reported histone acetyltransferase inhibitors. Nat. Commun. 8, 1527. 10.1038/s41467-017-01657-3 29142305 PMC5688144

[B19] Dou Y. Milne T. A. Ruthenburg A. J. Lee S. Lee J. W. Verdine G. L. (2006). Regulation of MLL1 H3K4 methyltransferase activity by its core components. Nat. Struct. Mol. Biol. 13, 713–719. 10.1038/nsmb1128 16878130

[B20] Early J. J. Cole K. L. Williamson J. M. Swire M. Kamadurai H. Muskavitch M. (2018). An automated high-resolution *in vivo* screen in zebrafish to identify chemical regulators of myelination. Elife 7, e35136. 10.7554/eLife.35136 29979149 PMC6056238

[B21] Elsalini O. A. Rohr K. B. (2003). Phenylthiourea disrupts thyroid function in developing zebrafish. Dev. Genes Evol. 212, 593–598. 10.1007/s00427-002-0279-3 12536323

[B22] Fasano C. A. Dimos J. T. Ivanova N. B. Lowry N. Lemischka I. R. Temple S. (2007). shRNA knockdown of Bmi-1 reveals a critical role for p21-Rb pathway in NSC self-renewal during development. Cell Stem Cell 1, 87–99. 10.1016/j.stem.2007.04.001 18371338

[B23] Feng W. Kawauchi D. Korkel-Qu H. Deng H. Serger E. Sieber L. (2017). Chd7 is indispensable for mammalian brain development through activation of a neuronal differentiation programme. Nat. Commun. 8, 14758. 10.1038/ncomms14758 28317875 PMC5364396

[B24] Filippi A. Durr K. Ryu S. Willaredt M. Holzschuh J. Driever W. (2007). Expression and function of nr4a2, lmx1b, and pitx3 in zebrafish dopaminergic and noradrenergic neuronal development. BMC Dev. Biol. 7, 135. 10.1186/1471-213X-7-135 18053265 PMC2217549

[B25] Filippi A. Jainok C. Driever W. (2012). Analysis of transcriptional codes for zebrafish dopaminergic neurons reveals essential functions of Arx and Isl1 in prethalamic dopaminergic neuron development. Dev. Biol. 369, 133–149. 10.1016/j.ydbio.2012.06.010 22728160

[B26] Gajer J. M. Furdas S. D. Grunder A. Gothwal M. Heinicke U. Keller K. (2015). Histone acetyltransferase inhibitors block neuroblastoma cell growth *in vivo* . Oncogenesis 4, e137. 10.1038/oncsis.2014.51 25664930 PMC4338425

[B27] Gregg R. G. Willer G. B. Fadool J. M. Dowling J. E. Link B. A. (2003). Positional cloning of the young mutation identifies an essential role for the Brahma chromatin remodeling complex in mediating retinal cell differentiation. Proc. Natl. Acad. Sci. U. S. A 100, 6535–6540. 10.1073/pnas.0631813100 12748389 PMC164481

[B28] Harrison I. F. Dexter D. T. (2013). Epigenetic targeting of histone deacetylase: therapeutic potential in Parkinson's disease? Pharmacol. Ther. 140, 34–52. 10.1016/j.pharmthera.2013.05.010 23711791

[B29] Harrison M. R. Georgiou A. S. Spaink H. P. Cunliffe V. T. (2011). The epigenetic regulator Histone Deacetylase 1 promotes transcription of a core neurogenic programme in zebrafish embryos. BMC Genomics 12, 24. 10.1186/1471-2164-12-24 21226904 PMC3032698

[B30] Hegarty S. V. Sullivan A. M. O'Keeffe G. W. (2013). Midbrain dopaminergic neurons: a review of the molecular circuitry that regulates their development. Dev. Biol. 379, 123–138. 10.1016/j.ydbio.2013.04.014 23603197

[B31] Higashijima S. Hotta Y. Okamoto H. (2000). Visualization of cranial motor neurons in live transgenic zebrafish expressing green fluorescent protein under the control of the islet-1 promoter/enhancer. J. Neurosci. 20, 206–218. 10.1523/JNEUROSCI.20-01-00206.2000 10627598 PMC6774115

[B32] Hirabayashi Y. Suzki N. Tsuboi M. Endo T. A. Toyoda T. Shinga J. (2009). Polycomb limits the neurogenic competence of neural precursor cells to promote astrogenic fate transition. Neuron 63, 600–613. 10.1016/j.neuron.2009.08.021 19755104

[B33] Holzschuh J. Ryu S. Aberger F. Driever W. (2001). Dopamine transporter expression distinguishes dopaminergic neurons from other catecholaminergic neurons in the developing zebrafish embryo. Mech. Dev. 101, 237–243. 10.1016/S0925-4773(01)00287-8 11231083

[B34] Holzschuh J. Hauptmann G. Driever W. (2003). Genetic analysis of the roles of Hh, FGF8, and nodal signaling during catecholaminergic system development in the zebrafish brain. J. Neurosci. 23, 5507–5519. 10.1523/JNEUROSCI.23-13-05507.2003 12843251 PMC6741235

[B35] Houzelstein D. Bullock S. L. Lynch D. E. Grigorieva E. F. Wilson V. A. Beddington R. S. P. (2002). Growth and early postimplantation defects in mice deficient for the bromodomain-containing protein Brd4. Mol. Cell. Biol. 22, 3794–3802. 10.1128/MCB.22.11.3794-3802.2002 11997514 PMC133820

[B36] Hsieh J. Zhao X. (2016). Genetics and Epigenetics in Adult Neurogenesis. Cold Spring Harb. Perspect. Biol. 8, a018911. 10.1101/cshperspect.a018911 27143699 PMC4888816

[B37] Jacob J. Ribes V. Moore S. Constable S. C. Sasai N. Gerety S. S. (2014). Valproic acid silencing of ascl1b/Ascl1 results in the failure of serotonergic differentiation in a zebrafish model of fetal valproate syndrome. Dis. Model Mech. 7, 107–117. 10.1242/dmm.013219 24135485 PMC3882053

[B38] Jacobs F. M. van Erp S. van der Linden A. J. von Oerthel L. Burbach J. P. Smidt M. P. (2009). Pitx3 potentiates Nurr1 in dopamine neuron terminal differentiation through release of SMRT-mediated repression. Development 136, 531–540. 10.1242/dev.029769 19144721

[B39] Jakovcevski M. Akbarian S. (2012). Epigenetic mechanisms in neurological disease. Nat. Med. 18, 1194–1204. 10.1038/nm.2828 22869198 PMC3596876

[B40] Jang M. K. Mochizuki K. Zhou M. Jeong H. S. Brady J. N. Ozato K. (2005). The bromodomain protein Brd4 is a positive regulatory component of P-TEFb and stimulates RNA polymerase II-dependent transcription. Mol. Cell 19, 523–534. 10.1016/j.molcel.2005.06.027 16109376

[B41] Kaslin J. Panula P. (2001). Comparative anatomy of the histaminergic and other aminergic systems in zebrafish (Danio rerio). J. Comp. Neurol. 440, 342–377. 10.1002/cne.1390 11745628

[B42] Kimmel C. B. Ballard W. W. Kimmel S. R. Ullmann B. Schilling T. F. (1995). Stages of embryonic development of the zebrafish. Dev. Dyn. 203, 253–310. 10.1002/aja.1002030302 8589427

[B43] Kokel D. Bryan J. Laggner C. White R. Cheung C. Y. Mateus R. (2010). Rapid behavior-based identification of neuroactive small molecules in the zebrafish. Nat. Chem. Biol. 6, 231–237. 10.1038/nchembio.307 20081854 PMC2834185

[B44] Korb E. Herre M. Zucker-Scharff I. Darnell R. B. Allis C. D. (2015). BET protein Brd4 activates transcription in neurons and BET inhibitor Jq1 blocks memory in mice. Nat. Neurosci. 18, 1464–1473. 10.1038/nn.4095 26301327 PMC4752120

[B45] Kubicek S. Gilbert J. C. Fomina-Yadlin D. Gitlin A. D. Yuan Y. Wagner F. F. (2012). Chromatin-targeting small molecules cause class-specific transcriptional changes in pancreatic endocrine cells. Proc. Natl. Acad. Sci. U. S. A. 109, 5364–5369. 10.1073/pnas.1201079109 22434908 PMC3325696

[B46] Lagger G. O'Carroll D. Rembold M. Khier H. Tischler J. Weitzer G. (2002). Essential function of histone deacetylase 1 in proliferation control and CDK inhibitor repression. EMBO J. 21, 2672–2681. 10.1093/emboj/21.11.2672 12032080 PMC126040

[B47] Lee S. Rellinger E. J. Kim K. W. Craig B. T. Romain C. V. Qiao J. (2015). Bromodomain and extraterminal inhibition blocks tumor progression and promotes differentiation in neuroblastoma. Surgery 158, 819–826. 10.1016/j.surg.2015.04.017 26067464 PMC4536146

[B48] Lim D. A. Huang Y. C. Swigut T. Mirick A. L. Garcia-Verdugo J. M. Wysocka J. (2009). Chromatin remodelling factor Mll1 is essential for neurogenesis from postnatal neural stem cells. Nature 458, 529–533. 10.1038/nature07726 19212323 PMC3800116

[B49] Lohr H. Ryu S. Driever W. (2009). Zebrafish diencephalic A11-related dopaminergic neurons share a conserved transcriptional network with neuroendocrine cell lineages. Development 136, 1007–1017. 10.1242/dev.033878 19234064

[B50] Loven J. Hoke H. A. Lin C. Y. Lau A. Orlando D. A. Vakoc C. R. (2013). Selective inhibition of tumor oncogenes by disruption of super-enhancers. Cell 153, 320–334. 10.1016/j.cell.2013.03.036 23582323 PMC3760967

[B51] Mahler J. Filippi A. Driever W. (2010). DeltaA/DeltaD regulate multiple and temporally distinct phases of notch signaling during dopaminergic neurogenesis in zebrafish. J. Neurosci. 30, 16621–16635. 10.1523/JNEUROSCI.4769-10.2010 21148001 PMC6634882

[B52] Mahmoud F. Tampi R. R. (2011). Valproic acid-induced parkinsonism in the elderly: a comprehensive review of the literature. Am. J. Geriatr. Pharmacother. 9, 405–412. 10.1016/j.amjopharm.2011.09.002 21993183

[B53] Marks P. A. Breslow R. (2007). Dimethyl sulfoxide to vorinostat: development of this histone deacetylase inhibitor as an anticancer drug. Nat. Biotechnol. 25, 84–90. 10.1038/nbt1272 17211407

[B54] Mertz J. A. Conery A. R. Bryant B. M. Sandy P. Balasubramanian S. Mele D. A. (2011). Targeting MYC dependence in cancer by inhibiting BET bromodomains. Proc. Natl. Acad. Sci. 108, 16669–16674. 10.1073/pnas.1108190108 21949397 PMC3189078

[B55] North T. E. Goessling W. Walkley C. R. Lengerke C. Kopani K. R. Lord A. M. (2007). Prostaglandin E2 regulates vertebrate haematopoietic stem cell homeostasis. Nature 447, 1007–1011. 10.1038/nature05883 17581586 PMC2775137

[B56] O'Carroll D. Erhardt S. Pagani M. Barton S. C. Surani M. A. Jenuwein T. (2001). The polycomb-group gene Ezh2 is required for early mouse development. Mol. Cell Biol. 21, 4330–4336. 10.1128/MCB.21.13.4330-4336.2001 11390661 PMC87093

[B57] Perino M. Veenstra G. J. (2016). Chromatin Control of Developmental Dynamics and Plasticity. Dev. Cell 38, 610–620. 10.1016/j.devcel.2016.08.004 27676434

[B58] Peterson R. T. Fishman M. C. (2011). “Chapter 23 - Designing Zebrafish Chemical Screens,” in Methods in Cell Biology, vol. 525-541 . Eds. Detrich H. W. Westerfield M. Zon L. I. (San Diego, California: Academic Press). 10.1016/B978-0-12-381320-6.00023-0 21951546

[B59] Peterson R. T. Link B. A. Dowling J. E. Schreiber S. L. (2000). Small molecule developmental screens reveal the logic and timing of vertebrate development. Proc. Natl. Acad. Sci. U. S. A 97, 12965–12969. 10.1073/pnas.97.24.12965 11087852 PMC27161

[B60] Pfefferli C. Muller F. Jazwinska A. Wicky C. (2014). Specific NuRD components are required for fin regeneration in zebrafish. BMC Biol. 12, 30. 10.1186/1741-7007-12-30 24779377 PMC4038851

[B61] Pinho B. R. Reis S. D. Guedes-Dias P. Leitao-Rocha A. Quintas C. Valentao P. (2016). Pharmacological modulation of HDAC1 and HDAC6 *in vivo* in a zebrafish model: Therapeutic implications for Parkinson's disease. Pharmacol. Res. 103, 328–339. 10.1016/j.phrs.2015.11.024 26657418

[B62] Renani P. G. Taheri F. Rostami D. Farahani N. Abdolkarimi H. Abdollahi E. (2019). Involvement of aberrant regulation of epigenetic mechanisms in the pathogenesis of Parkinson's disease and epigenetic-based therapies. J. Cell Physiol. 234, 19307–19319. 10.1002/jcp.28622 30968426

[B63] Richter S. Schulze U. Tomancak P. Oates A. C. (2017). Small molecule screen in embryonic zebrafish using modular variations to target segmentation. Nat. Commun. 8, 1901. 10.1038/s41467-017-01469-5 29196645 PMC5711842

[B64] Rihel J. Prober D. A. Arvanites A. Lam K. Zimmerman S. Jang S. (2010). Zebrafish behavioral profiling links drugs to biological targets and rest/wake regulation. Science 327, 348–351. 10.1126/science.1183090 20075256 PMC2830481

[B65] Rink E. Wullimann M. F. (2002). Development of the catecholaminergic system in the early zebrafish brain: an immunohistochemical study. Brain Res. Dev. Brain Res. 137, 89–100. 10.1016/S0165-3806(02)00354-1 12128258

[B66] Robertson A. L. Holmes G. R. Bojarczuk A. N. Burgon J. Loynes C. A. Chimen M. (2014). A zebrafish compound screen reveals modulation of neutrophil reverse migration as an anti-inflammatory mechanism. Sci. Transl. Med. 6, 225ra229. 10.1126/scitranslmed.3007672 PMC4247228 24574340

[B67] Rodriguez P. Bonte E. Krijgsveld J. Kolodziej K. E. Guyot B. Heck A. J. (2005). GATA-1 forms distinct activating and repressive complexes in erythroid cells. EMBO J. 24, 2354–2366. 10.1038/sj.emboj.7600702 15920471 PMC1173143

[B68] Ryu S. Mahler J. Acampora D. Holzschuh J. Erhardt S. Omodei D. (2007). Orthopedia homeodomain protein is essential for diencephalic dopaminergic neuron development. Curr. Biol. 17, 873–880. 10.1016/j.cub.2007.04.003 17481897

[B69] Ryu Y. K. Park H. Y. Go J. Kim Y. H. Hwang J. H. Choi D. H. (2018). Effects of histone acetyltransferase inhibitors on L-DOPA-induced dyskinesia in a murine model of Parkinson's disease. J. Neural Transm. (Vienna) 125, 1319–1331. 10.1007/s00702-018-1902-4 29998409

[B70] Sallinen V. Torkko V. Sundvik M. Reenila I. Khrustalyov D. Kaslin J. (2009). MPTP and MPP+ target specific aminergic cell populations in larval zebrafish. J. Neurochem. 108, 719–731. 10.1111/j.1471-4159.2008.05793.x 19046410

[B71] San B. Chrispijn N. D. Wittkopp N. van Heeringen S. J. Lagendijk A. K. Aben M. (2016). Normal formation of a vertebrate body plan and loss of tissue maintenance in the absence of ezh2. Sci. Rep. 6, 24658. 10.1038/srep24658 27145952 PMC4857124

[B72] Sato Y. Hilbert L. Oda H. Wan Y. Heddleston J. M. Chew T. L. (2019). Histone H3K27 acetylation precedes active transcription during zebrafish zygotic genome activation as revealed by live-cell analysis. Development, 146, dev179127. 10.1242/dev.179127 31570370 PMC6803375

[B73] Schumacher A. Lichtarge O. Schwartz S. Magnuson T. (1998). The murine Polycomb-group gene eed and its human orthologue: functional implications of evolutionary conservation. Genomics 54, 79–88. 10.1006/geno.1998.5509 9806832

[B74] Stadler J. A. Shkumatava A. Norton W. H. Rau M. J. Geisler R. Fischer S. (2005). Histone deacetylase 1 is required for cell cycle exit and differentiation in the zebrafish retina. Dev. Dyn. 233, 883–889. 10.1002/dvdy.20427 15895391

[B75] Stern H. M. Murphey R. D. Shepard J. L. Amatruda J. F. Straub C. T. Pfaff K. L. (2005). Small molecules that delay S phase suppress a zebrafish bmyb mutant. Nat. Chem. Biol. 1, 366–370. 10.1038/nchembio749 16372403

[B76] van Heesbeen H. J. Smidt M. P. (2019). Entanglement of Genetics and Epigenetics in Parkinson's Disease. Front. Neurosci. 13, 277. 10.3389/fnins.2019.00277 30983962 PMC6449477

[B77] van Heesbeen H. J. Mesman S. Veenvliet J. V. Smidt M. P. (2013). Epigenetic mechanisms in the development and maintenance of dopaminergic neurons. Development 140, 1159–1169. 10.1242/dev.089359 23444349

[B78] Vleeshouwer-Neumann T. Phelps M. Bammler T. K. MacDonald J. W. Jenkins I. Chen E. Y. (2015). Histone deacetylase inhibitors antagonize distinct pathways to suppress tumorigenesis of embryonal rhabdomyosarcoma. PloS One 10, e0144320. 10.1371/journal.pone.0144320 26636678 PMC4670218

[B79] Westerfield M. (2000). The zebrafish book. A guide for the laboratory use of zebrafish (Danio rerio). 4th ed. (Eugene: Univ. of Oregon Press).

[B80] Wever I. von Oerthel L. Wagemans C. Smidt M. P. (2018). EZH2 Influences mdDA neuronal differentiation, maintenance and survival. Front. Mol. Neurosci. 11, 491. 10.3389/fnmol.2018.00491 30705619 PMC6344421

[B81] Yamamoto K. Vernier P. (2011). The evolution of dopamine systems in chordates. Front. Neuroanat. 5, 21. 10.3389/fnana.2011.00021 21483723 PMC3070214

[B82] Yao B. Christian K. M. He C. Jin P. Ming G. L. Song H. (2016). Epigenetic mechanisms in neurogenesis. Nat. Rev. Neurosci. 17, 537–549. 10.1038/nrn.2016.70 27334043 PMC5610421

[B83] Yeh J. R. Crews C. M. (2003). Chemical genetics: adding to the developmental biology toolbox. Dev. Cell 5, 11–19. 10.1016/S1534-5807(03)00200-4 12852848

[B84] Yu B. D. Hess J. L. Horning S. E. Brown G. A. Korsmeyer S. J. (1995). Altered Hox expression and segmental identity in Mll-mutant mice. Nature 378, 505–508. 10.1038/378505a0 7477409

