# Supplementary material for: Chemical Genetics Screen Identifies Epigenetic Mechanisms Involved in Dopaminergic and Noradrenergic Neurogenesis in Zebrafish
Source: Front Genet. 2020 Feb 25;11:80. doi: 10.3389/fgene.2020.00080 (PMC7052299; doi:10.3389/fgene.2020.00080)
Supplement: Supplementary file 1 [file DataSheet_1.pdf]

Supplementary Material

Chemical genetics screen identifies epigenetic mechanisms involved in dopaminergic and noradrenergic neurogenesis in zebrafish

Markus Westphal, Pooja Sant, Alexander-Thomas Hauser, Manfred Jung and Wolfgang Driever

Inventory of Supplementary Materials:

Supplementary Figures 1 through 7

Supplementary Figures 1: Overview of small molecule inhibitors of chromatin regulators tested.
Supplementary Figures 2: Examples for small molecule compound treatments that cause no change in DA and NA marker expression.
Supplementary Figures 3: Effects of selected HDAC, Bromodomain and HAT inhibitor treatments on DA and NA marker expression when applied during different developmental time windows.
Supplementary Figures 4: Secondary Screen of HDAC, Bromodomain, HAT and MLL1-WDR5 small molecule inhibitors.
Supplementary Figures 5: Quantification of Secondary Screen of MLL1-WDR5 inhibitors.
Supplementary Figures 6: Evaluation of potential developmental delay: Determination of retinal diameter in HDAC, Bromodomain, HAT or MLL1-WDR5 inhibitor treated zebrafish embryos.
Supplementary Figures 7: Phospho-Histone H3 immunohistochemistry.

Supplementary References

Primary data from chemical screens

Supplementary Table 1 - Primary screen compounds with DA/NA phenotypes 1-30 µM
Supplementary Table 2 - Primary screen compounds 100 µM
Supplementary Table 3 - Primary screen compounds no DA/NA phenotypes 1-30 µM
Supplementary Table 4 - Secondary screen DA/NA cell counts
Supplementary Table 5 - Secondary screen *isl1* cell counts
Supplementary Table 6 - Secondary screen *sox2* phenotypes
Supplementary Table 7 - Secondary screen apoptosis cell counts
Supplementary Table 8 - Secondary screen retina diameter measurements

Target Family	Target	Small molecule inhibitor	Supplier	References
Histone deacetylases (HDAC)	pan-HDAC	Vorinostat	CRC992 MedEP	Richon et al., 1998
		SW55	CRC992 MedEP	Morera et al., 2016
	HDAC Class 1	Entinostat	CRC992 MedEP	Saito et al., 1999
		Mocetinostat	CRC992 MedEP; Selleckchem #S1122	Fournel et al., 2008
	HDAC6/10	JS28 (cpd4g)	CRC992 MedEP	Senger et al., 2016
		JS08 (cpd4b)	CRC992 MedEP	Senger et al., 2016
Sirtuins	Sirtuin1	EX-527	Selleckchem #S1541	Gertz et al., 2013
	Sirtuin 2	AGK-1	CRC992 MedEP	Outeiro et al., 2007
Histone acetyl-transferases (HAT)	pan-HAT	PU139	CRC992 MedEP	Gajer et al. 2015
		PU141	CRC992 MedEP	Gajer et al., 2015
		C646	CRC992 MedEP	Bowers et al., 2010
	pan-HAT/ LSD1	Curcumin	CRC992 MedEP	Balasubramanyam et al., 2004
Bromodomain	pan-Bromodomain	Bromosporine	CRC992 MedEP; Sigma #SML0992	Picaud et al., 2016
	BRD4, BRD2, BRD3	I-Bet151	CRC992 MedEP	Dawson et al., 2011
		JQ1	CRC992 MedEP	Fillippakopoulos et al., 2010
	CREBBP/EP300	SGC-CBP30	CRC992 MedEP	Hay et al., 2014
	BRD7, BRD9	LP99	SGC	Clark et al., 2015
	BRPF1, BRPF2, BRPF3	OF-1	SGC	http://www.thesgc.org/chemical-probes/OF-1
	BRPF1, BRPF2, BRPF3	NI-57	SGC	http://www.thesgc.org/chemical-probes/NI-57
	SMARCA2, SMARCA4	PFI-3	Selleckchem #S7315	Fedorov et al., 2015
Histone lysine methyl-transferases	Dot1l	SGC-0946	CRC992 MedEP	Yu et al., 2012
	SETD7	PFI-2	CRC992 MedEP	Barsyte-Lovejoy et al., 2014
	EZH2	GSK343	CRC992 MedEP	Verma et al., 2012
	MLL1-WDR5	MM-102	Selleckchem #S7265	Karatas et al., 2013
	MLL1-WDR5	OICR9429	SGC; Tocris # 5267	Grebrien et al., 2015
	G9a	UNC0631	Selleckchem, #S7610	Liu et al., 2011
	SMYD2	LLY-507	SGC	Nguyen et al., 2015
Histone lysine demethylases	LSD1	Tranylcypromine	CRC992 MedEP	Lee et al., 2006
		Namoline	CRC992 MedEP	Willmann et al., 2012
		CBB1007	CRC992 MedEP	Wang et al., 2011
	JmJ	Deferasirox	CRC992 MedEP	Roatsch et al., 2019
Histone-lysine reader	L3MBTL3	UNC1215	CRC992 MedEP	James et al., 2013

Supplementary Figure 1: Overview of small molecule inhibitors of chromatin regulators tested

CRC992 MedEp: Collaborative Reseach Centre Medical Epigenetics; https://www.sfb992.uni-freiburg.de/

SGC: Structural Genomics Consortium; https://www.thesgc.org/

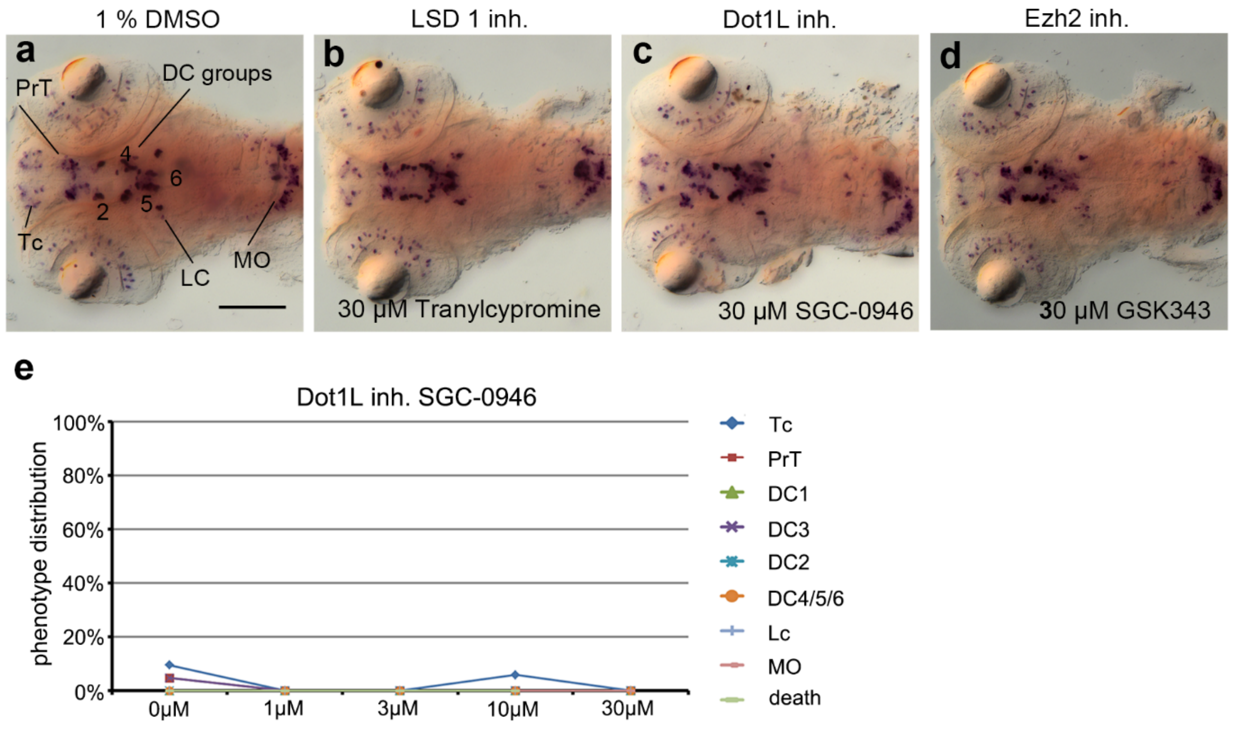

Supplementary Figure 2: Examples for small molecule compound treatments that cause no change in DA and NA marker expression.

(a-d) Whole mount *in situ* hybridization for expression of *th* as DA and NA neuron marker in embryos fixed at 86 hpf**. (a)** 1%DMSO treated control embryo. **(b-d)** Embryos treated with inhibitors as indicated. **(e)** Dose response curve for the Dot1L inhibitor SGC-0496 representing a small molecule compound that causes no change in DA and NA marker expression. Each line represents a selected DA or NA neuron cluster in which an effect was observed. Each data point represents the percentage of embryos showing an effect in the respective neuron cluster at the depicted concentration. **(a,e)** Abbreviations:, Tc: telencephalon, PrT: pretectum, DC1-6: diencephalic DA groups 1-6, Lc: locus coeruleus, MO: medulla oblongata.

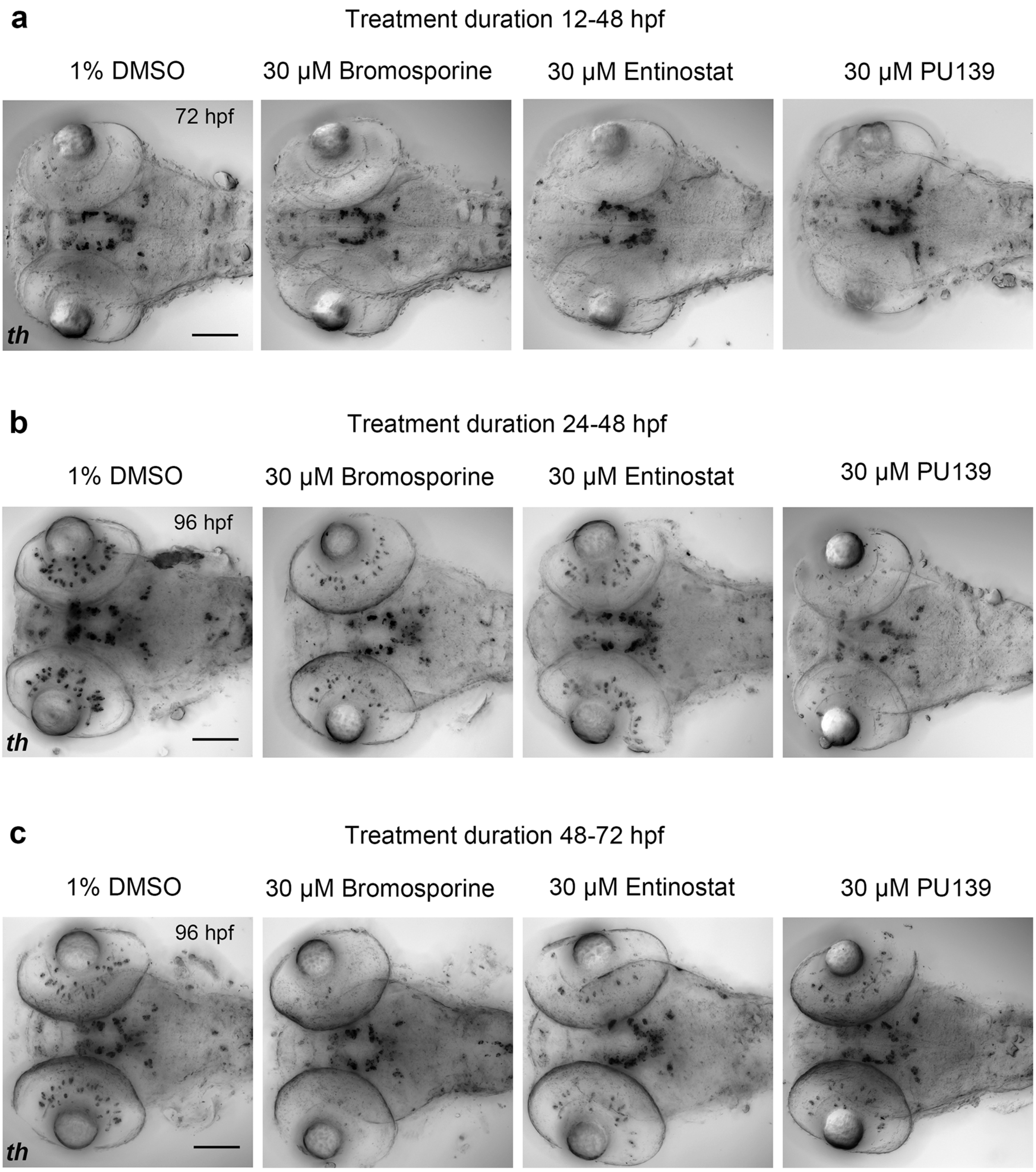

Supplementary Figure 3: Effects of selected HDAC, Bromodomain and HAT inhibitor treatments on DA and NA marker expression when applied during different developmental time windows.

(a-c) Images of embryos analyzed by whole mount *in situ* hybridization for expression of *th* as marker for DA and NA neurons. Duration of inhibitor treatments as indicated. **(a)** Embryos fixed at 72 hpf after Bromodomain inhibitor Bromosporine, HDAC inhibitor Entinostat or HAT inhibitor PU139 treatments as indicated. (**b,c)** Embryos fixed at 96 hpf after Bromodomain, HDAC and HAT inhibitor treatments as indicated. Images show dorsal views of head generated from Z-Projections from image stacks. Scale bars in left panel for whole row represent 100µm.

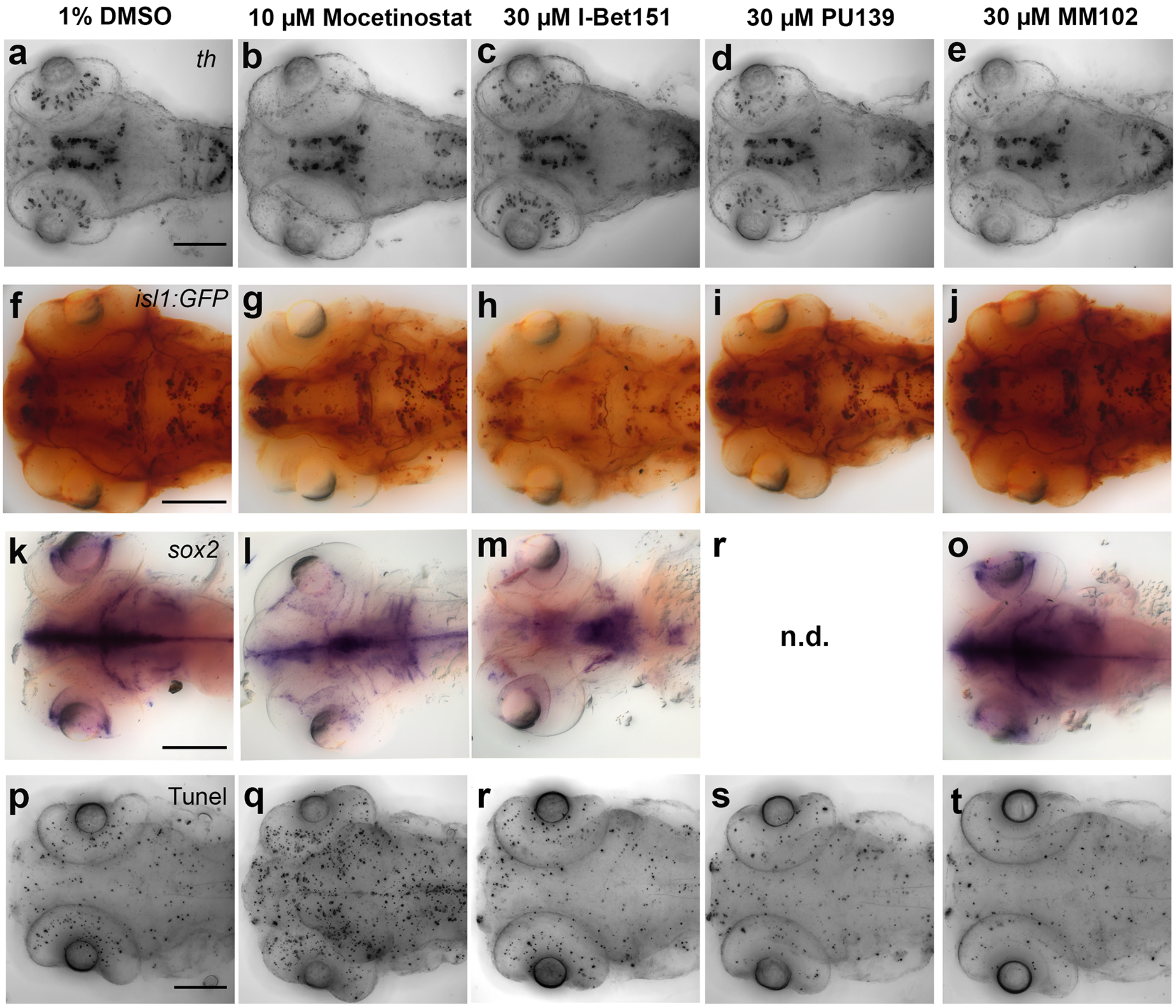

Supplementary Figure 4: Secondary Screen of HDAC, Bromodomain, HAT and MLL1-WDR5 small molecule inhibitors.

(**a-t**) Analysis of neuronal and cell death markers following treatments with DMSO control or inhibitors as indicated at top of each column of image panels. (**a-e**) Images of whole mount *in situ* hybridization for *th* as a DA and NA neuron marker. **(f-j)** whole mount immunohistochemistry for GFP in Tg(*isl1:GFP*)$^{rw0/+}$ embryos, which marks cranial motor neurons. **(k-o)** whole mount *in situ* hybridization for *sox2* as a neural stem cell marker. **(p-t)** and TUNEL assay. All embryos were treated from 24-72 hpf with compounds as indicated, and fixed at 96 hpf. Images show dorsal views of head generated from Z-Projections from image stacks. Scale bars in left panel for whole row represent 100µm.

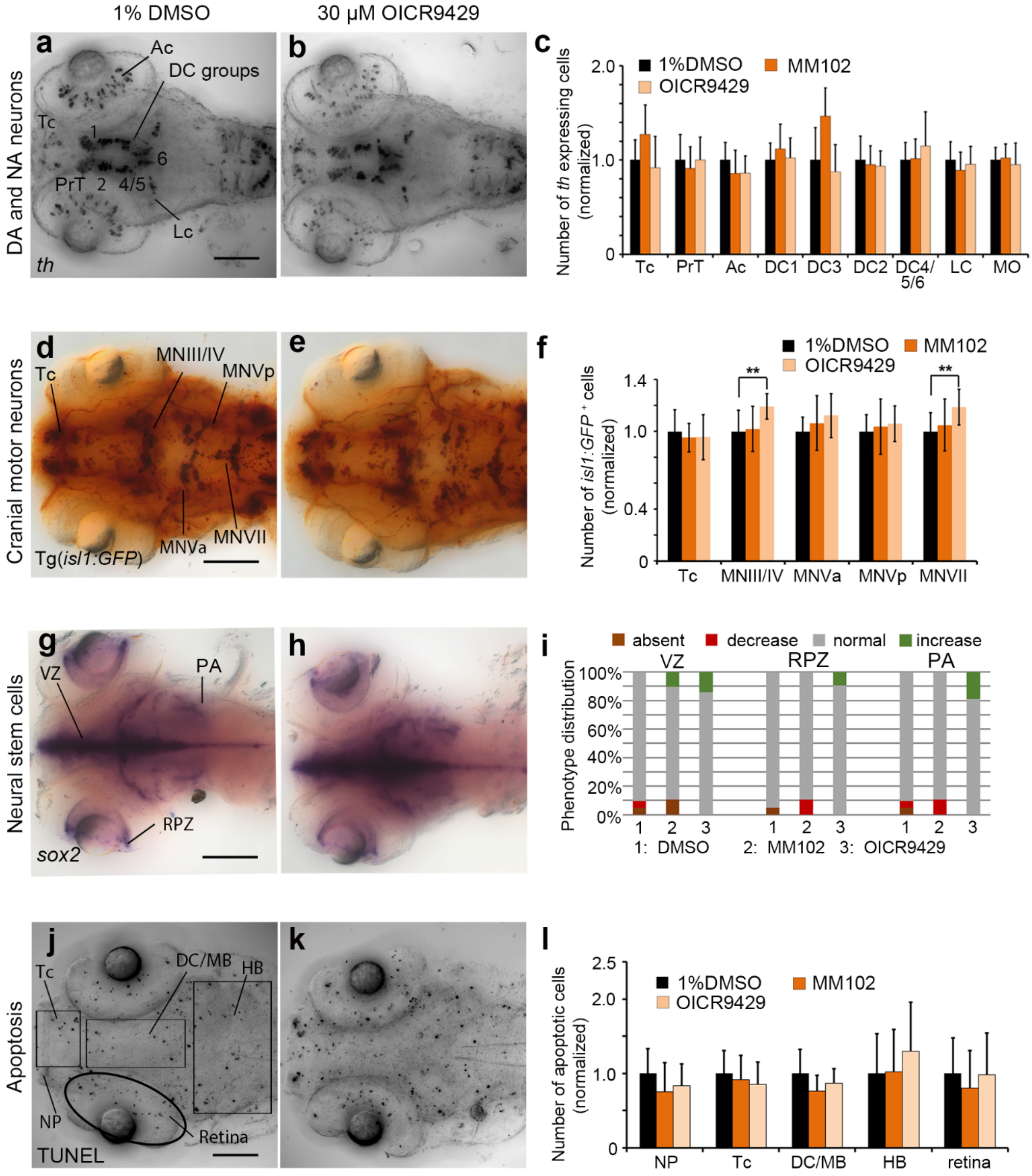

Supplementary Figure 5: Quantification of Secondary Screen of MLL1-WDR5 inhibitors.

(a-c) WISH analysis for DA and NA neuron development. **(d-f)** Immunohistochemistry for cranial motor neurons. **(g-i)** WISH analysis of *sox2* expression in neural stem cells. **(j-l)** Tunel assay to detect apoptotic cells. All embryos were treated with the MLL1-WDR5 interaction inhibitors MM102 or OICR9429as indicated from 24-72 hpf and fixed at 96 hpf. **(a,b,c,d,g,h,j,k)** Images show dorsal views of heads generated from Z-projections of image stacks. Scale bars represent 100µm. Bar charts illustrate the mean cell count numbers of each neuronal subtype for **(c)** *th* expressing cells, **(f)** *isl1:GFP* transgene expressing cells, **(l)** apoptotic cells. Error bars depict standard deviations of the means. Asterisks indicate significant differences compared with the 1% DMSO control ($p<0.001$). **(i)** For *sox2* expression, embryos were classified into absent, decreased, normal or increased *sox2* expression phenotypes (see color code) and embryo numbers normalized to 100%. Abbreviations: AC amacrine cells, DA – dopaminergic, NA - noradrenergic, Tc telencephalon, PrT pretectum, DC diencephalic groups, Lc locus coeruleus, MO/AP medulla oblongata/area postrema, MN motor neuron cluster, VZ ventricular zone, RPZ retinal proliferation zone, PA pharyngeal arches, NP: nasal placodes, DC/MB: diencephalon & midbrain region, DC: diencephalon, HB: hindbrain.

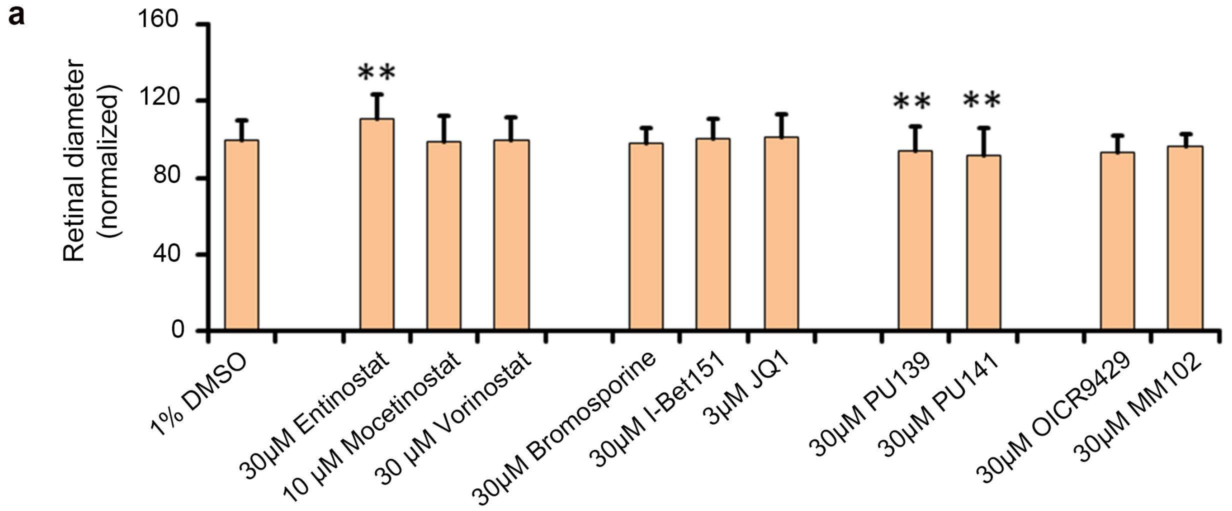

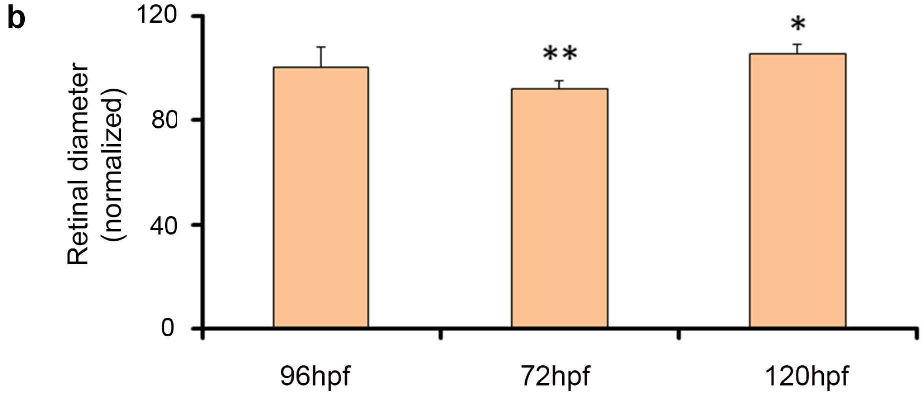

Supplementary Figure 6: Evaluation of potential developmental delay: Determination of retinal diameter in HDAC, Bromodomain, HAT or MLL1-WDR5 inhibitor treated zebrafish embryos.

(a) Bar chart representing the average retinal diameter in selected HDAC, Bromodomain, HAT and MLL1-WDR5 inhibitor treated zebrafish embryos as well as 1% DMSO treated controls. The retinal diameter was measured in embryos fixed measured using photographs embryos fixed at 96 hpf. The retinal diameter measurements average of control embryos was set to 100, and all measurements of treated embryos normalized with respect to this value. **(b)** Bar chart representing the average retinal diameter in WT zebrafish embryos at 96 hpf, 72 hpf and 120 hpf. The retinal diameter measurements average of 96 hpf embryos was set to 100, and measurements of 72 and 120 hpf embryos normalized with respect to this value. **(a,b)** Error bars depict standard deviation of the mean. Asterisks indicate significant differences (*p<0.05 and **p<0,001) compared to control embryos treated to the 1% DMSO control **(a)** or the WT retina at 96 hpf **(b)**.

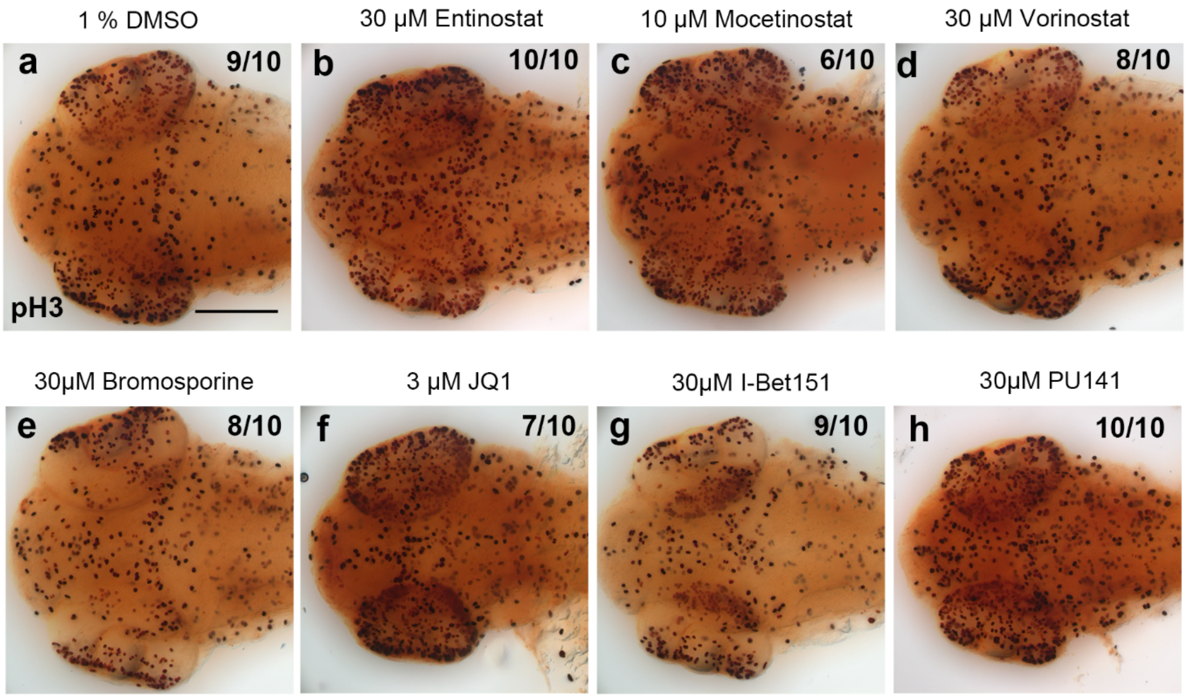

Supplementary Figure 7: Phospho-Histone H3 immunohistochemistry.

(a-h) Distribution of the mitotic marker phospho-Histone 3 (pH3) detected at 72 hpf by whole mount immunohistochemistry in embryos fixed after drug or control treatments. Treatments are indicated above each panel. Dorsal views of heads of larvae, images generated from Z-Projections of image stacks. Scale bar in (a) is 100 µm for all panels. Numbers indicate representative phenotype and total embryos analyzed.

Supplementary References

Balasubramanyam K, Varier RA, Altaf M, Swaminathan V, Siddappa NB, Ranga U, et al. Curcumin, a novel p300/CREB-binding protein-specific inhibitor of acetyltransferase, represses the acetylation of histone/nonhistone proteins and histone acetyltransferase-dependent chromatin transcription. J Biol Chem (2004) 279**:** 51163-51171.

Barsyte-Lovejoy D, Li F, Oudhoff MJ, Tatlock JH, Dong A, Zeng H, et al. (R)-PFI-2 is a potent and selective inhibitor of SETD7 methyltransferase activity in cells. Proc Natl Acad Sci U S A (2014) 111**:** 12853-12858.

Bowers EM, Yan G, Mukherjee C, Orry A, Wang L, Holbert MA, et al. Virtual ligand screening of the p300/CBP histone acetyltransferase: identification of a selective small molecule inhibitor. Chem Biol (2010) 17**:** 471-482.

Clark PG, Vieira LC, Tallant C, Fedorov O, Singleton DC, Rogers CM, et al. LP99: Discovery and Synthesis of the First Selective BRD7/9 Bromodomain Inhibitor. Angew Chem Int Ed Engl (2015) 54**:** 6217-6221.

Dawson MA, Prinjha RK, Dittmann A, Giotopoulos G, Bantscheff M, Chan WI, et al. Inhibition of BET recruitment to chromatin as an effective treatment for MLL-fusion leukaemia. Nature (2011) 478**:** 529-533.

Fedorov O, Castex J, Tallant C, Owen DR, Martin S, Aldeghi M, et al. Selective targeting of the BRG/PB1 bromodomains impairs embryonic and trophoblast stem cell maintenance. Sci Adv (2015) 1**:** e1500723.

Filippakopoulos P, Qi J, Picaud S, Shen Y, Smith WB, Fedorov O, et al. Selective inhibition of BET bromodomains. Nature (2010) 468**:** 1067-1073.

Fournel M, Bonfils C, Hou Y, Yan PT, Trachy-Bourget MC, Kalita A, et al. MGCD0103, a novel isotype-selective histone deacetylase inhibitor, has broad spectrum antitumor activity in vitro and in vivo. Mol Cancer Ther (2008) 7**:** 759-768.

Gajer JM, Furdas SD, Grunder A, Gothwal M, Heinicke U, Keller K, et al. Histone acetyltransferase inhibitors block neuroblastoma cell growth in vivo. Oncogenesis (2015) 4**:** e137.

Gertz M, Fischer F, Nguyen GT, Lakshminarasimhan M, Schutkowski M, Weyand M, et al. Ex-527 inhibits Sirtuins by exploiting their unique NAD+-dependent deacetylation mechanism. Proc Natl Acad Sci U S A (2013) 110**:** E2772-2781.

Grebien F, Vedadi M, Getlik M, Giambruno R, Grover A, Avellino R, et al. Pharmacological targeting of the Wdr5-MLL interaction in C/EBPalpha N-terminal leukemia. Nat Chem Biol (2015) 11**:** 571-578.

Hay DA, Fedorov O, Martin S, Singleton DC, Tallant C, Wells C, et al. Discovery and optimization of small-molecule ligands for the CBP/p300 bromodomains. J Am Chem Soc (2014) 136**:** 9308-9319.

James LI, Barsyte-Lovejoy D, Zhong N, Krichevsky L, Korboukh VK, Herold JM, et al. Discovery of a chemical probe for the L3MBTL3 methyllysine reader domain. Nat Chem Biol (2013) 9**:** 184-191.

Karatas H, Townsend EC, Cao F, Chen Y, Bernard D, Liu L, et al. High-affinity, small-molecule peptidomimetic inhibitors of MLL1/WDR5 protein-protein interaction. J Am Chem Soc (2013) 135**:** 669-682.

Lee MG, Wynder C, Schmidt DM, McCafferty DG, Shiekhattar R. Histone H3 lysine 4 demethylation is a target of nonselective antidepressive medications. Chem Biol (2006) 13**:** 563-567.

Liu F, Barsyte-Lovejoy D, Allali-Hassani A, He Y, Herold JM, Chen X, et al. Optimization of cellular activity of G9a inhibitors 7-aminoalkoxy-quinazolines. J Med Chem (2011) 54**:** 6139-6150.

Morera L, Roatsch M, Fürst MCD, Hoffmann I, Senger J, Hau M, et al. 4-Biphenylalanine- and 3-Phenyltyrosine-Derived Hydroxamic Acids as Inhibitors of the JumonjiC-Domain-Containing Histone Demethylase KDM4A. ChemMedChem (2016) 11**:** 2063-2083.

Nguyen H, Allali-Hassani A, Antonysamy S, Chang S, Chen LH, Curtis C, et al. LLY-507, a Cell-active, Potent, and Selective Inhibitor of Protein-lysine Methyltransferase SMYD2. J Biol Chem (2015) 290**:** 13641-13653.

Outeiro TF, Kontopoulos E, Altmann SM, Kufareva I, Strathearn KE, Amore AM, et al. Sirtuin 2 inhibitors rescue alpha-synuclein-mediated toxicity in models of Parkinson's disease. Science (2007) 317**:** 516-519.

Picaud S, Leonards K, Lambert JP, Dovey O, Wells C, Fedorov O, et al. Promiscuous targeting of bromodomains by bromosporine identifies BET proteins as master regulators of primary transcription response in leukemia. Sci Adv (2016) 2**:** e1600760.

Richon VM, Emiliani S, Verdin E, Webb Y, Breslow R, Rifkind RA, et al. A class of hybrid polar inducers of transformed cell differentiation inhibits histone deacetylases. Proc Natl Acad Sci U S A (1998) 95**:** 3003-3007.

Roatsch M, Hoffmann I, Abboud MI, Hancock RL, Tarhonskaya H, Hsu KF, et al. The Clinically Used Iron Chelator Deferasirox Is an Inhibitor of Epigenetic JumonjiC Domain-Containing Histone Demethylases. ACS Chem Biol (2019) 14**:** 1737-1750.

Saito A, Yamashita T, Mariko Y, Nosaka Y, Tsuchiya K, Ando T, et al. A synthetic inhibitor of histone deacetylase, MS-27-275, with marked in vivo antitumor activity against human tumors. Proc Natl Acad Sci U S A (1999) 96**:** 4592-4597.

Senger J, Melesina J, Marek M, Romier C, Oehme I, Witt O, et al. Synthesis and Biological Investigation of Oxazole Hydroxamates as Highly Selective Histone Deacetylase 6 (HDAC6) Inhibitors. J Med Chem (2016) 59**:** 1545-1555.

Verma SK, Tian X, LaFrance LV, Duquenne C, Suarez DP, Newlander KA, et al. Identification of Potent, Selective, Cell-Active Inhibitors of the Histone Lysine Methyltransferase EZH2. ACS Med Chem Lett (2012) 3**:** 1091-1096.

Wang J, Lu F, Ren Q, Sun H, Xu Z, Lan R, et al. Novel histone demethylase LSD1 inhibitors selectively target cancer cells with pluripotent stem cell properties. Cancer Res (2011) 71**:** 7238-7249.

Willmann D, Lim S, Wetzel S, Metzger E, Jandausch A, Wilk W, et al. Impairment of prostate cancer cell growth by a selective and reversible lysine-specific demethylase 1 inhibitor. Int J Cancer (2012) 131**:** 2704-2709.

Yu W, Chory EJ, Wernimont AK, Tempel W, Scopton A, Federation A, et al. Catalytic site remodelling of the DOT1L methyltransferase by selective inhibitors. Nat Commun (2012) 3**:** 1288.
